# Advances in Double‐Stranded DNA Targeting Technologies

**DOI:** 10.1002/EXP.20250065

**Published:** 2026-02-02

**Authors:** Zuhao Shen, Yiqun Liu, Yingjie Hao, Yifan Bo, Xiaochuan Dai, Shihui Wang, Tian Xia, Xin Su, Huiyu Liu

**Affiliations:** ^1^ State Key Laboratory of Organic‐Inorganic Composites Beijing Key Laboratory of Bioprocess Beijing Advanced Innovation Center for Soft Matter Science and Engineering College of Life Science and Technology Beijing University of Chemical Technology Beijing China; ^2^ School of Biomedical Engineering Tsinghua University Beijing China; ^3^ David Geffen School of Medicine University of California Los Angeles California USA

**Keywords:** artificial Intelligence, double‐stranded DNA, gene targeting, gene editing, in situ Imaging, in vitro detection

## Abstract

Double‐stranded DNA (dsDNA) serves as a fundamental repository of genetic information and plays a pivotal role in the diagnosis and therapeutic management of diseases. However, the inherent stability of the DNA double helix under physiological conditions presents a challenge in accessing internal bases. To address this, various molecular targeting technologies have been developed, offering high specificity while destabilizing the DNA structure. This review provides a comprehensive overview of current dsDNA targeting tools, such as hybridization probes, modified nucleic acid probes, zinc finger proteins (ZFPs), transcription activator‐like effector nucleases (TALENs), the CRISPR/Cas system, Argonaute proteins (Agos), and the lambda exonuclease‐pDNA system (λ Exo‐pDNA), and some cutting‐edge molecular tools. It delves into the mechanisms behind these technologies. It highlights their applications in diverse areas, including in vitro detection, in situ imaging, gene editing, and their integration with artificial intelligence (AI)‐driven tools. Additionally, the review compares these techniques, discusses future technological opportunities, and identifies challenges in integrating these tools into diagnostic and therapeutic practices. By providing a holistic view of these rapidly evolving technologies, this review aims to fill a gap in the current literature and explore the future potential of dsDNA targeting innovations.

## Graphical abstract

Double‐stranded DNA (dsDNA) plays a crucial role in genetic information storage and disease management, but its inherent stability limits access to internal bases. To overcome this challenge, various high‐specificity molecular targeting technologies have been developed to destabilize the DNA structure. This review summarizes existing dsDNA‐targeting tools, their mechanisms, and applications in gene detection, imaging, and editing, while also exploring future directions and challenges.

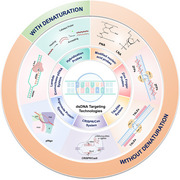

## 1 Introduction

Seventy years ago, James Watson and Francis Crick proposed the double‐helix model of DNA [1], based on X‐ray diffraction images of DNA crystals taken by Rosalind Franklin. This discovery founded modern molecular biology. It reshaped how we understand genetic information storage and transmission. Genes, the fundamental carriers of hereditary information, are typically encoded as double‐stranded DNA (dsDNA). The double‐helix structure of DNA elucidates how genetic material is stably stored and precisely replicated, driven by several synergistic factors: (1) Specific hydrogen bonding between base pairs [1, 2], where adenine (A) pairs with thymine (T) via two hydrogen bonds, and guanine (G) pairs with cytosine (C) via three hydrogen bonds; (2) π–π aromatic interactions between adjacent bases [3, 4] enhances the helix's compactness and stability; and (3) the hydration layer surrounding the double helix maintains its three‐dimensional architecture [5, 6, 7, 8]. The structural stability of DNA ensures the reliable storage and transmission of genetic information. However, the tightly intertwined double‐stranded structure and its spatial barriers often hinder direct access to the target sequence.

Direct and specific dsDNA targeting is highly significant in biomedicine, particularly in disease diagnosis and treatment. Targeting technologies can screen nucleic acid biomarkers relevant to diseases in genetic detection. For instance, in cancer diagnostics, detecting cancer marker sequences like *EGFR* [9, 10, 11], *KRAS* [12, 13], and *ALK* [14, 15] provides crucial information for tumor profiling. This helps with early detection, precision treatment, and monitoring therapeutic responses. In the study of bacterial resistance, screening bacterial genomic mutations aids in identifying drug resistance mechanisms [16, 17], and offers critical insights for developing antimicrobial strategies. Moreover, advances in dsDNA targeting technologies have facilitated the potential for precision therapy. The applications of gene editing tools, such as CRISPR/Cas9 [18, 19], hold promise for correcting pathogenic mutations and repairing genetic defects [20, 21, 22, 23]; dsDNA targeting technologies can also be used in agricultural production to enhance crop traits, improve resistance to diseases and pests, and increase yield [24, 25, 26, 27]. By precisely editing plant genomes, these technologies enable the development of crops with better nutritional profiles, tolerance to environmental stresses, and improved growth efficiency.

**FIGURE 1:**
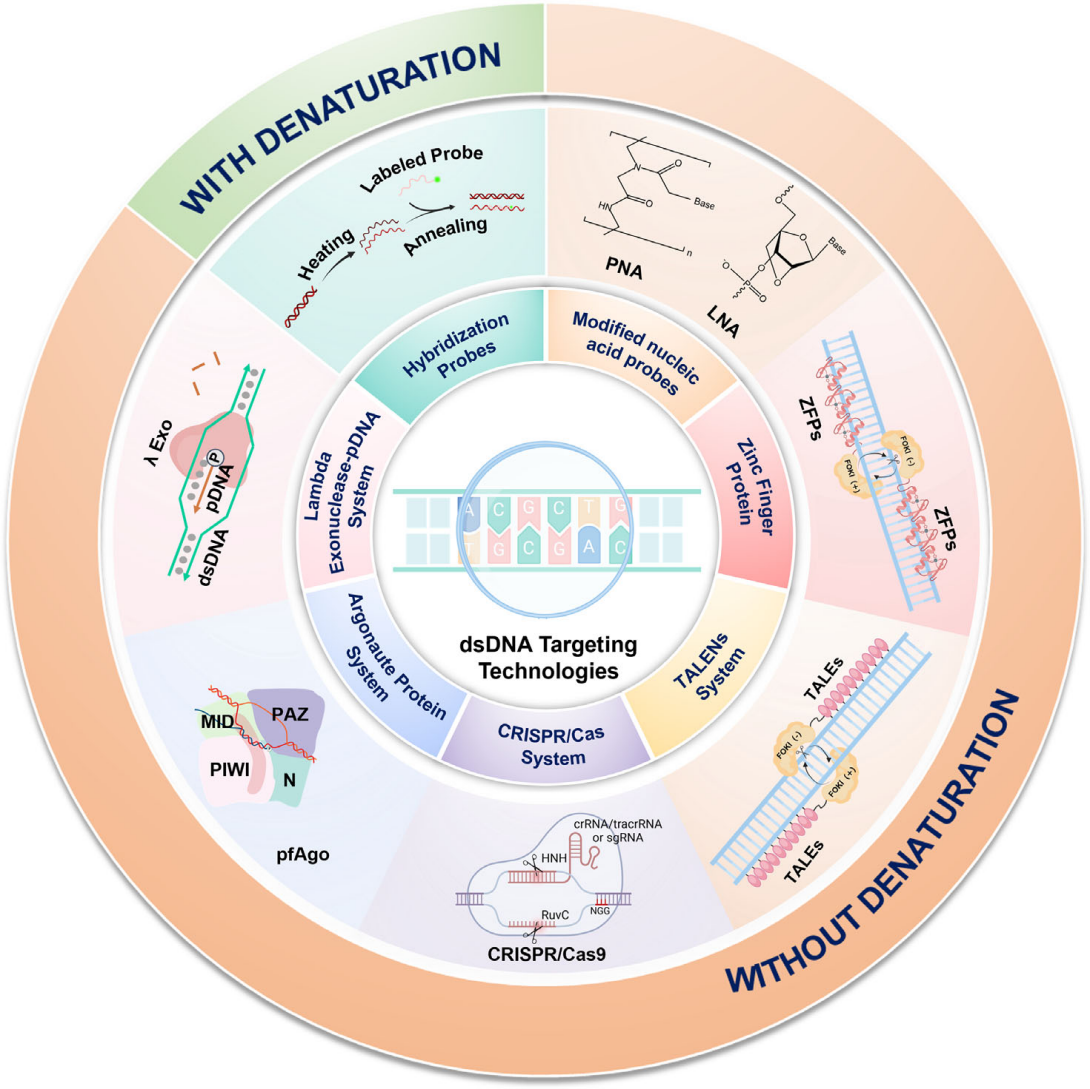
Overview of dsDNA technologies.

The concept of gene targeting first emerged in the 1980s. Over the past four decades, scientists have explored natural systems and developed a variety of molecular targeting tools using physicochemical approaches. This review explores molecular tools for precise dsDNA targeting, presenting them in chronological order while grouping them based on technological similarity. We begin with denaturation‐dependent strategies, such as normal hybridization probes, followed by denaturation‐independent protein‐free approaches, including PNA and LNA. We then introduce denaturation‐independent protein‐based systems, such as zinc finger proteins and TALENs, CRISPR/Cas systems, Argonaute protein systems, and the lambda Exonuclease‐pDNA system. Additionally, we discuss some cutting‐edge molecular tools (Figure 1). We outline the fundamental principles of each system, followed by an analysis of how its physical and biological properties contribute to achieving high specificity in dsDNA targeting. Additionally, we examine the applications of these technologies based on their unique characteristics. The review concludes with a discussion of the challenges associated with these methods and provides perspectives on their future development. By offering a comprehensive overview, this review aims to drive the advancement of dsDNA targeting technologies in both fundamental research and practical applications, with their integration with AI‐driven tools to enhance performance.

## 2 Denaturation‐Dependent dsDNA Targeting Strategies

### 2.1 Denaturation‐Enabled dsDNA Targeting and Advances in FISH

**FIGURE 2:**
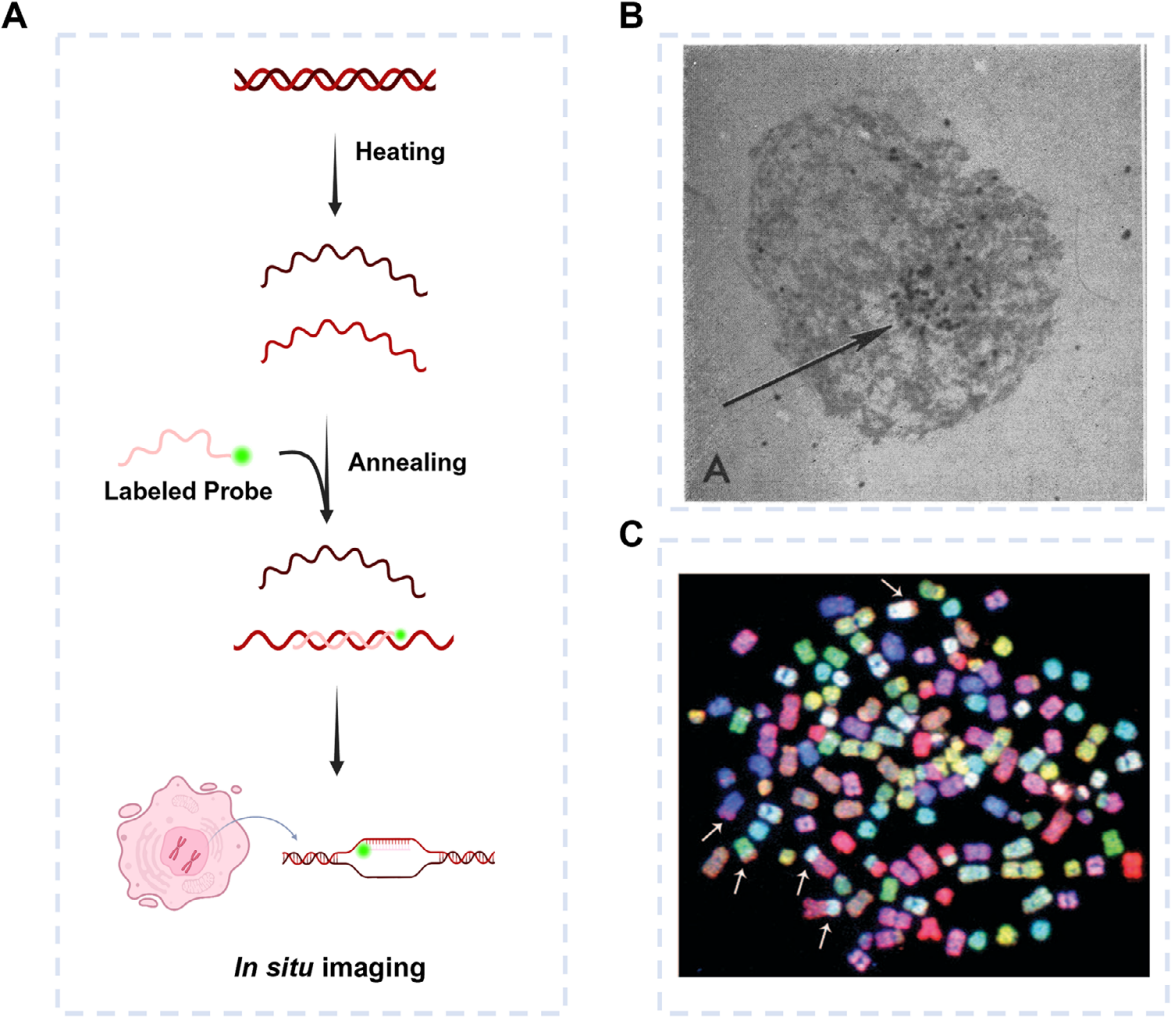
Hybridization probes for gene in situ imaging. (A) Schematic of hybridization probes and in situ imaging. (B) Radioisotope‐labeled RNA detection in African clawed toad oocytes to study DNA transcription. Reproduced with permission [39]. Copyright 1969, National Academy of Sciences. (C) Metaphase spread from a human bladder cancer cell line hybridized with a SKY probe. Reproduced with permission [40]. Copyright 2006, Springer Nature.

Hybridization probes are nucleic acid detection tools whose specificity is based on the principles of Watson–Crick base pairing. The thermodynamic stability of duplexes formed by perfectly complementary strands is markedly higher than that of those containing mismatches, thereby ensuring enhanced specificity and sensitivity in detection. In general, a single nucleotide mismatch can lead to a reduction in the melting temperature (*T*_m_) of approximately 1–5°C. They can serve as primers in various gene amplification systems, including polymerase chain reaction (PCR) and quantitative real‐time PCR (RT‐qPCR). Additionally, these probes can be labeled with fluorescent groups and quenchers for direct gene detection. With the rapid advancement of DNA nanotechnology [28, 29, 30], these hybridization probes have evolved into various forms and reaction types, including toehold‐mediated strand displacement (TMSD) [31, 32], hybridization chain reaction (HCR) [32, 33, 34], and catalytic hairpin assembly (CHA) [32, 35, 36], etc. However, due to the inherent stability of the DNA double helix, the hybridization probes face difficulty invading the dsDNA substrate and initiating subsequent recognition reactions. For effective hybridization, the dsDNA substrate must first be denatured into two ssDNAs (Figure 2A). This process is typically achieved through heating or chemical denaturants [37, 38]. Heat denaturation of DNA occurs as increased molecular motion disrupts hydrogen bonds and weakens π–π stacking, leading to strand separation. Chemical denaturation, by contrast, relies on denaturants that compete for base binding, alter solvent conditions, and disrupt base stacking and hydration. However, the efficiency of chemical denaturation depends on the type, concentration, and exposure time of the denaturant, and it is often incompatible with downstream applications. As a result, thermal denaturation is generally preferred for its simplicity and precise temperature control.

The advancement of in situ hybridization technology has gone hand in hand with innovations in hybridization probes, with both driving significant progress in genomic detection technologies. As early as the 1960s, Joseph and Mary proposed a molecular hybridization technique to locate DNA sequences within chromosomes. In 1969, they detected complementary DNA sequences in the frog egg genome by denaturing the DNA with alkali, followed by hybridization with tritiated RNA [39]. The DNA–RNA hybridized complexes were then detected using radioautography (Figure 2B). This pioneering research laid the foundation for in situ gene detection at the cellular level. By the 1970s, Rudkin et al. [41] pioneered fluorescence in situ hybridization (FISH), using indirect immunofluorescence to detect DNA‐RNA hybrids, where fluorescent groups replaced radioactive elements. As fluorescence technology advanced, multicolor FISH [42] and spectral karyotyping (SKP) [40] (Figure 2C) were introduced in the 1990s. These methods enable multiple fluorescent probes with different colors to identify various chromosomes or regions within a single chromosome. FISH can be used not only to determine chromosome locations but also to detect abnormalities such as duplications, deletions, translocations, and other chromosomal alterations.

With the continuous advancement of molecular biology and the breakthroughs in super‐resolution microscopy [43, 44, 45, 46, 47], various advanced FISH technologies have emerged, including single‐molecule FISH (smFISH) [48], microautoradiography FISH (MAR‐FISH) [49], spatial transcriptomics analysis via RNA‐FISH (STARFISH) [50], multiplexed error‐robust FISH (MERFISH) [51, 52, 53], etc. These methods offer distinct imaging advantages for different scenarios and have advanced the study of spatial transcriptomics, particularly in brain gene imaging. For example, MERFISH [51] developed by Xiaowei Zhuang's team, is a high‐throughput spatial transcriptomics technology for spatial localization analysis of gene expression at the single‐cell level. Its principle assigns each target RNA a unique binary barcode, read bit by bit using fluorescent probes in multiple hybridization rounds. Microscopy captures the signals to decode the RNA's identity and position in cells, with error‐correcting codes ensuring high accuracy.

It is noteworthy that mRNA is commonly employed in these advanced FISH approaches. DNA loci need to be labeled and transcribed into RNA to be imaged (e.g., in Zhuang's work) indirectly. Direct imaging of genomic DNA using normal hybridization probes under denaturation‐free conditions remains challenging. This difficulty can be addressed by combining FISH with protein‐based molecular tools, such as CRISPR‐FISH and lambda‐FISH, which will be discussed in subsequent sections.

### 2.2 Helper DNA‐Assisted Hybridization

**FIGURE 3:**
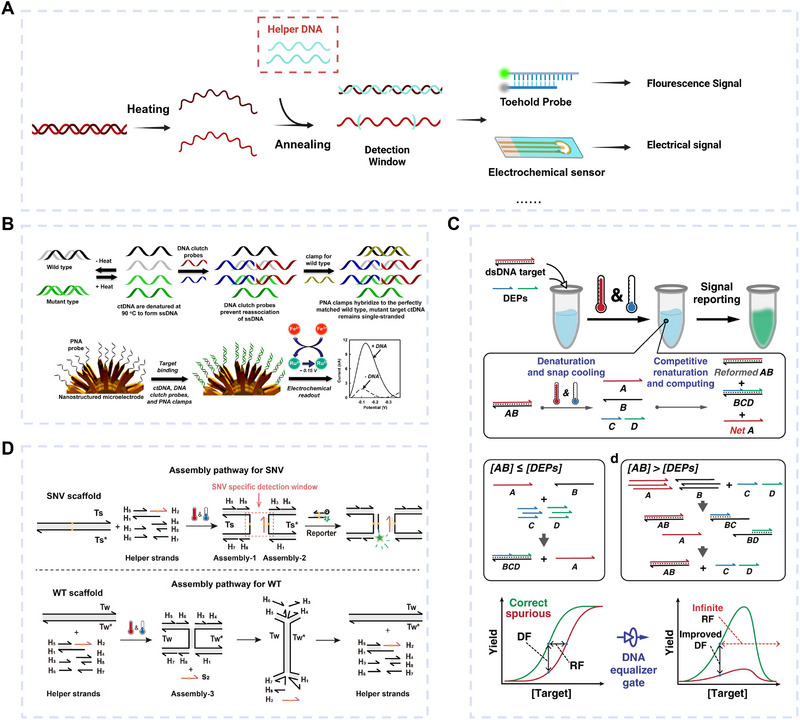
Gene detection based on Helper DNA‐assisted hybridization. (A) Schematic diagram of Helper DNA‐assisted hybridization. (B) DCPs enhance the capture efficiency of hybridization probes on nanoelectrodes using Helper DNA strands. Reproduced with permission [54]. Copyright 2016, American Chemical Society. (C) DNA clutch probes employ Helper DNA strands to design hybridization probes that expand the detection window. Reproduced with permission [55]. Copyright 2020, Springer Nature. (D) PANDA utilizes six Helper DNA strands to eliminate interference caused by wild‐type sequences. Reproduced with permission [56]. Copyright 2023, American Chemical Society.

Utilizing helper DNA strands to facilitate probe binding to the target gene is an effective strategy. The Helper DNA, typically a single‐stranded DNA (ssDNA) strand, is designed based on thermodynamic principles to partially complement the target sequence, essentially functioning as a hybridization probe. After denaturation and annealing, the Helper DNA first competitively hybridizes with the target DNA, forming a stable complex that stabilizes the double‐helix structure and prevents the reannealing of target ssDNA strands. As a result, the detection window of the target DNA is exposed, allowing hybridization probes to bind directly and trigger a signal response (Figure 3A). The specificity of this targeting relies on both the reporter probe and the Helper DNA, with the latter also enabling precise discrimination of nucleic acid mutations in the target DNA through careful sequence design. For instance, Song et al. [54] developed an electrochemical assay called DNA clutch probes (DCPs, Figure 3B) for analyzing circulating tumor DNA (ctDNA). The DCPs were a pair of ssDNA molecules that prevented the target ssDNA from recombining during annealing. This enabled the detection window to hybridize to a probe immobilized on a nano‐microelectrode, allowing the detection of mutated ctDNA as low as 0.01%. By analyzing the mutation status of ctDNA in the serum samples from nine lung cancer patients (*KRAS* mutations) and nine melanoma patients (*BRAF* mutations), the assay eventually obtained consistent results with clamp PCR. Similarly, Feng Li's team designed DNA equalizer probes (DEPs) with Helper DNA by simulation (Figure 3C), achieving a wider detection window. In their study, two Helper DNA strands competitively hybridized with the single‐stranded region of dsDNA, thereby exposing the detection window for interaction with the TMSD probe labeled with a fluorophore and a quencher. They integrated DEPs with PCR to form DEG‐PCR, determining mutations by analyzing the difference in the fluorescence signal profiles generated by wild‐type (WT) and mutant‐type (MT) targets. DEPs achieved a LOD of 1 aM and could differentiate mismatches at 1 pM and perfect matches at 10 aM. Utilizing DEG‐PCR to detect soil‐transmitted helminths samples excreted from six children in Honduras, the results were consistent with NGS sequencing results. Although DCPs and DEPs have demonstrated superior advantages and detection limits in distinguishing single nucleotide variants (SNVs), they are prone to false positive results. The specificity of double helper DNA is still limited to a certain extent, especially under conditions of high WT concentration or less stringent reaction conditions. To address the false positive, Feng Li's team proposed post amplification SNV‐specific DNA assembly (PANDA, Figure 3D) [56]. Through the meticulous design of seven helper strands based on thermodynamic principles, PANDA effectively mitigated the pressure brought by high concentrations of WT genes. PANDA detected the *EGFR* gene variant allele frequency (VAF) as low as 0.1%. Employing PANDA to quantify *EGFR* mutant genes in plasma samples from 68 patients and pleural exudate samples from four patients, the analyses were in high agreement with the results of next‐generation sequencing.

Overall, denaturation‐dependent strategies provide a universal dsDNA targeting approach but face challenges such as precise temperature control, probe efficiency fluctuations, and non‐specific binding risks. Advances like molecular dynamics simulation‐based probe design and deep learning signal analysis can be employed to enhance specificity and accuracy.

## 3 Denaturation‐Independent dsDNA Targeting: Probing Native dsDNA Structures

Denaturation‐independent dsDNA targeting strategies eliminate the need to convert dsDNA into ssDNA. Instead, these strategies allow probes to directly and specifically bind to the target gene sequence, enabling probing of native dsDNA structure. By bypassing the denaturation process, these strategies prevent potential DNA damage or structural alterations, offering a gentler approach to recognition. Current denaturation‐independent targeting technologies can be broadly categorized into two categories: (1) protein‐free recognition methods, including modified nucleic acid probes, and (2) protein‐involved strategies, such as Zinc finger DNA binding proteins (ZFPs), transcription activator‐like effector nucleases (TALENs), CRISPR/Cas system, Argonaute protein, and lambda exonuclease‐pDNA system. This section will systematically review their principles, applications, and recent developments.

### 3.1 Modified Nucleic Acid Probes: Peptide Nucleic Acid and Locked Nucleic Acid

**FIGURE 4:**
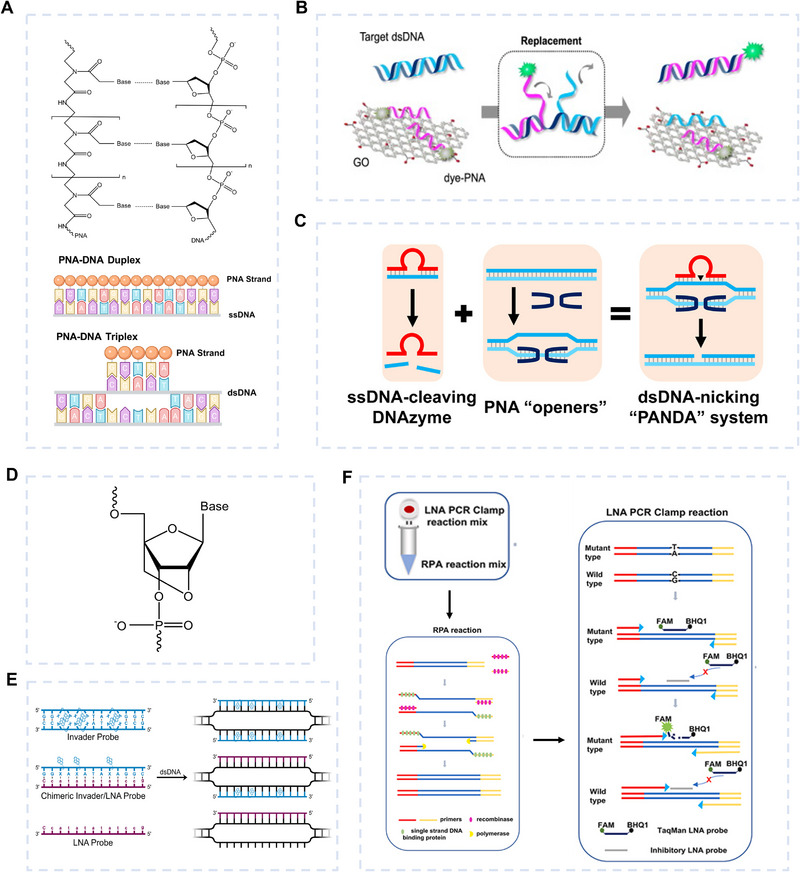
dsDNA targeting methods based on modified nucleic acid probes. (A–C) Peptide nucleic acid probes for dsDNA targeting. (A) Chemical structure of PNA. (B) Direct detection of dsDNA targets using PNA and graphene oxide. Reproduced with permission [57]. Copyright 2014, Elsevier. (C) PNA strands are used as “openers” to invade dsDNA and expose ssDNA regions, followed by DNAzyme cleavage to generate double‐strand breaks (DSBs). Reproduced with permission [58]. Copyright 2021, American Chemical Society. (D–F) Locking nucleic acid probes for dsDNA targeting. (D) Chemical structure of LNA. (E) LNA invader probe forms a triplex structure with dsDNA. Reproduced with permission [59]. Copyright 2024, Royal Society of Chemistry. (F) The RLP technique, combining RPA and LNA probes, enables the detection of the *EGFR* T790M mutation. Reproduced with permission [60]. Copyright 2024, Elsevier.

Peptide nucleic acid (PNA) was invented in 1991 by Peter E. Nielsen and colleagues [61]. Unlike DNA or RNA, PNA has a peptide backbone composed of *N*‐(2‐aminoethyl) glycine units, making it electrically neutral [62] (Figure 4A). The base pairing mechanism of PNA is the same as that of conventional DNA molecules [63, 64]. This neutral charge reduces electrostatic repulsion with DNA, allowing for stronger binding. In many cases, PNA can bind directly to dsDNA, either forming a triplex structure or disrupting hydrogen bonds, often without requiring denaturation [58, 65, 66, 67, 68, 69]. For example, Lee et al. [57] developed a dsDNA sensor using fluorescence‐labeled PNA and graphene oxide quenchers (Figure 4B). Since dsDNA ends tend to dissociate, PNA first binds to these ends. Its strong affinity triggers branch migration, displacing one DNA strand and forming a PNA/DNA duplex, which restores fluorescence. The hepatitis A virus Vall7 polyprotein gene (*HVA*), *HIV*, and hepatitis B virus surface antigen gene (*HVB*) were chosen as dsDNA targets, with a LOD of 400 pM. PNA could also be employed in FISH, allowing for gene imaging, provided that the non‐denaturation condition. For example, Pochechueva et al. [70] designed quantitative FISH (Q‐FISH) employing PNA probes to label telomeric DNA. Combined with 3D stimulated emission depletion (STED) nanoscopy, Q‐FISH enabled high‐resolution imaging of individual telomeres. PNA can also be used as an auxiliary strand in gene editing tools. Lee et al. [58] fabricated a system called PNA‐assisted double‐stranded DNA nicking by DNAzymes (PANDA) (Figure 4C), proposing a novel gene editing approach. PANDA used PNA as “openers” to invade dsDNA and expose ssDNA regions by forming base pairs with the complementary strand. Then, DNAzymes were employed to cleave specific sites on the ssDNA, resulting in double‐strand breaks DSBs in the dsDNA. The resulting dsDNA fragments can then be recombined with other fragments to facilitate DNA recombination.

Unlike PNA, which replaces the entire phosphate backbone, locked nucleic acid (LNA) modifies only the ribose. Introduced by Poul Nielsen's team in 1998 [71], LNA features a methylene bridge between the 2' and 4' carbons, locking the sugar in a stable C3'‐endo conformation (Figure 4D). This structural modification allows LNA to exhibit a higher binding affinity than conventional nucleic acid probes when hybridizing with nucleic acid molecules, including ssDNA, dsDNA, or ssRNA. Specifically, for each LNA monomer incorporated, the melting temperature of the resulting hybrid can increase by an average of 3–8°C. LNA probes can also directly access dsDNA in a denaturation‐independent system. For example, the LNA Invader probes invented by Patrick J. Hrdlicka's team [59, 72, 73] can selectively bind to telomeric DNA on chromosomes at temperatures significantly lower than the denaturation temperature (Figure 4E). Based on the strong affinity properties of LNA probes, Moreno et al. [74] designed bisLNA probes with a clamp structure that can invade supercoiled dsDNA through both Hoogsteen [75, 76, 77] and Watson–Crick base pairing. Huang et al. [60] exhibited a recombinase polymerase amplification (RPA)‐assisted LNA probe‐mediated dual amplification biosensing platform (RLP) (Figure 4F) for detecting the *EGFR T790M* mutation. They physically combined RPA and an improved LNA clamp PCR reaction into a single reaction, in which two rounds of amplification of the target region were carried out. During this process, the dual LNA probes used in the LNA clamp PCR reaction specifically targeted the target mutation, achieving a VAF of 0.007% in simulated serum samples.

Overall, PNA and LNA probes possess distinct molecular properties and application advantages. The high affinity and resistance to enzymatic degradation of PNA and LNA make them promising for a broad range of applications in precision gene detection. However, PNA suffers from poor cellular uptake and unfavorable pharmacokinetics [78], while high synthesis costs and low bioavailability limit LNA. In the future, the application scope of these modified probes could be expanded by leveraging their combined strengths or by developing novel nucleic acid modifications.

### 3.2 Zinc Finger DNA Binding Proteins (ZFPs)

Zinc finger DNA‐binding proteins (ZFPs) were initially identified in 1985 by Aaron Klug's team [79] while they were extensively studying the transcriptional regulation mechanisms in *Xenopus laevis* oocytes. These proteins are characterized by their modular finger‐like domains, each coordinating with zinc ions, with every zinc finger motif recognizing a specific triplet of nucleotides. Certain amino acids interact with specific bases through hydrogen bonding or van der Waals forces, as is shown in Table 1.

**TABLE 1:** Amino acids for base preference and interacting forces.

Amino acid	Base preference	Interacting force
Arg	G	Hydrogen bond
Asn/Gln	A	Hydrogen bond
Ser/Thr	T	Hydrogen bond
Asp/Glu	C	Hydrogen bond
His	G/C	Hydrogen bond π–π aromatic stacking
Lys	Phosphate backbone	Electrostatic attraction, Hydrogen bonding

**FIGURE 5:**
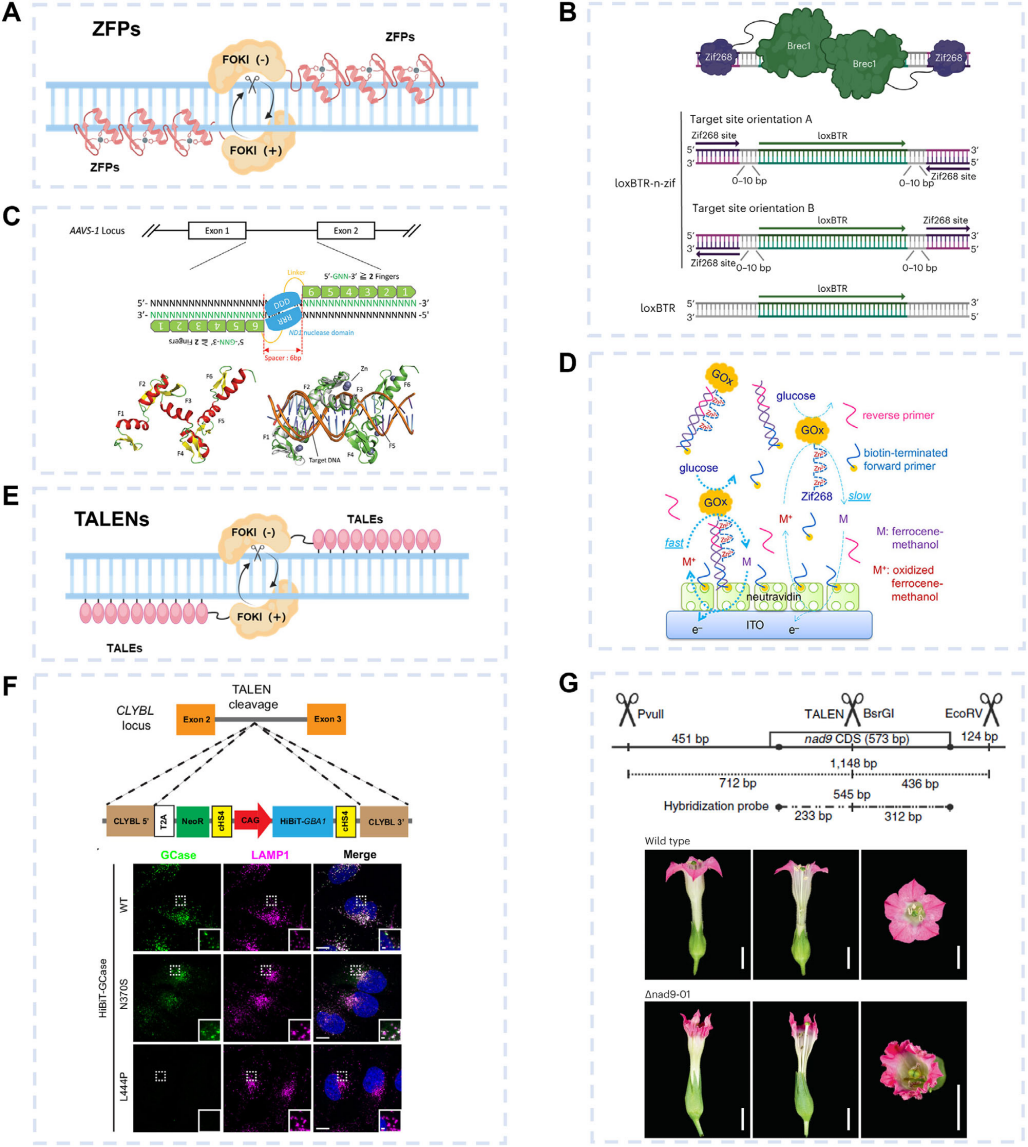
dsDNA targeting methods based on ZFPs and TALENs. (A–D) ZFPs for dsDNA targeting. (A) Schematic of ZFPs. (B) ZFDs to program Brec1 recombinase binding by developing fusions, and target site library overview. Reproduced with permission [85]. Copyright 2024, Springer Nature. (C) Engineering of ZFPs through structural modeling improves genome editing efficiency. Reproduced with permission [89]. Copyright 2024, Wiley. (D) Washing‐free electrochemical detection method based on ZFPs for detecting dsDNA amplified by RPA. Reproduced with permission [90]. Copyright 2018, American Chemical Society. (E–G) TALENs for dsDNA targeting. (E) Schematic of TALENs. (F) Utilizing TALENs stably integrates a HiBiT‐tagged GBA1 transgene in a GBA1 knockout H4 cell line, and colocalization of GCase (green) with lysosomal marker LAMP1 (magenta) was determined by immunofluorescent staining. Reproduced with permission [95]. Copyright 2024, National Academy of Sciences. (G) Targeted mutagenesis of the nad9 gene in plant mtDNA using TALENs, and the flower phenotype of nad9 knockout and wild‐type plants. Reproduced with permission [96, 97]. Copyright 2022 and 2023, Springer Nature.

The Cys_2_‐His_2_ zinc finger motif, such as Zif 268 [80], is widely used. It binds to DNA by inserting its α‐helix into the major groove of the double helix, forming a tripartite structure. Based on this characteristic, ZFPs were the first programmable nucleases, created by fusing zinc finger motifs with the FokI endonuclease for site‐specific DNA cleavage [81] (Figure 5A). To function, two ZFPs must recognize a 5–7 bp spacer region, each binding to opposite strands. This allows FokI monomers to dimerize and cut the DNA precisely.

ZFPs can also be fused with various enzymes for gene knockout [82, 83], knock‐in [84], or recombination [85], making ZFPs a versatile tool in the first generation of gene editing techniques [86, 87]. Recently, Mukhametzyanova et al. [85] harnessed zinc‐finger DNA‐binding domains to program Brec1 recombinase binding by developing fusions, in which Zif268 is inserted into Brec1 recombinase coding sequences (Figure 5B). In this fusion protein, Zif268 acts as a switch to activate the recombinase, which only triggers the gene editing reaction when at a specific site. This improved editing efficiency fourfold while reducing off‐target effects. Various AI tools like AlphaFold [88] are powerful platforms capable of accurately predicting protein–protein and protein‐nucleic acid interactions. For example, Katayama et al. [89] utilized AlphaFold [88] to assist in the engineering modification of ZFPs, resulting in a 5% increase in genome editing efficiency (Figure 5C). Of course, ZFPs can also be applied in gene detection. Fang et al. [90] developed a washing‐free electrochemical detection method based on ZFPs for detecting dsDNA amplified by RPA (Figure 5D). They achieved the differentiation between electrode‐bound and unbound tags by combining glucose oxidase with Zif268. This process utilizes proximity‐dependent electron mediation via ferrocenemethanol, eliminating the need for additional washing or incubation steps. The entire detection process was completed within 17 min, with a LOD of 300 copies/13.2 µL. Besides, the team led by Moon‐Soo Kim has conducted extensive research on using ZFPs for nucleic acid detection. Their work initially focused on the use of ZFP arrays for the visual detection of bacterial genomic DNA [91, 92] with a LOD of approximately 50 fM, later immobilizing ZFPs on polymer chips [93, 94], achieving LODs of ∼100 fM [93] and ∼10 fM [94]. These technologies have demonstrated exceptional specificity and sensitivity.

ZFPs, as first‐generation gene‐editing tools, offer several advantages, including high specificity, a broad targeting range, versatile functions, and a wealth of application experience. However, they also present challenges like complex design, off‐target effects, low editing efficiency, and high costs. To address these limitations, future improvements could focus on optimizing the design process, integrating innovative technologies, enhancing both specificity and efficacy, reducing costs, and broadening application areas. These advancements may fully unlock the potential of ZFPs in gene editing and nucleic acid detection.

### 3.3 Transcription Activator‐Like Effector Nucleases (TALENs)

TALENs originate from TALE proteins in *Xanthomonas* spp., which naturally regulate gene expression by binding specific DNA sequences. Unlike ZFPs, where each domain recognizes a triplet of bases, TALE repeat units bind to individual nucleotides, enhancing specificity. However, a key limitation is the requirement for the target site to begin with a T base, significantly restricting the range of selectable target sequences. Inspired by the design principles of ZFP‐based gene‐editing enzymes, researchers engineered a fusion of TALEs with the FokI nuclease in 2009. This innovation led to the development of TALENs, which emerged as the second‐generation, highly efficient gene‐editing tool [98, 99] (Figure 5E). For example, Williams et al. [95] used TALENs to precisely edit the *GBA1* gene, which encodes β‐glucocerebrosidase (GCase) (Figure 5F). They integrated a hybrid bacterial‐insect bioluminescence reporter (HiBiT) peptide tag at a specific “safe harbor” site in the genome of *GBA1* knockout glioma cell lines. This strategy facilitated the stable expression of *GBA1* variants with the HiBiT tag in the cells. The HiBiT tag could bind with high affinity to an exogenous luminescent bacterial bioluminescence peptide tag, thereby reconstructing an active luciferase enzyme. This enabled bioluminescent readouts of GCase protein levels in the cells, providing a sensitive and dynamic measurement of gene expression. Ralph Bock's team [96, 97] developed TALEN‐based gene drive mutation (TALEN‐GDM), which enables targeted mutagenesis of plant mitochondrial DNA (mtDNA). By designing TALENs targeting the tobacco mitochondrial *NADH dehydrogenase subunit 9* (*nad9*) gene, the researchers introduced DSBs in the mitochondrial genome (Figure 5G). They then screened for mutations resistant to TALEN cleavage, facilitated by a homologous recombination repair mechanism. This approach increased the mutation recovery rate and produced mutant plants capable of stably passing the edited mtDNA to their offspring. TALENs provide higher precision than ZFPs by recognizing single nucleotides [100].

Recent advances suggest emerging applications in gene detection. TALENs' unique ability to recognize extended DNA sequences (14–20 bp) without PAM restrictions makes them particularly suitable for detecting single‐nucleotide polymorphisms (SNPs) and epigenetic modifications [101, 102, 103]. For instance, TALE‐based molecular probes have been engineered for real‐time visualization of telomeric repeats and centromeric satellite DNA in live cells [112]. While current implementations remain less prevalent than CRISPR‐based detection systems, ongoing developments in split‐TALE reporter systems and modular scaffold designs show promise for creating highly specific biosensors [104]. These developments highlight TALENs' untapped potential in diagnostic applications, particularly in scenarios requiring long recognition sequences or PAM‐free targeting.

Until 2012, ZFPs and TALENs dominated dsDNA targeting, but their complexity, off‐target effects, and high engineering costs limited their applications. The emergence of CRISPR/Cas in 2012 revolutionized the field with its simplicity and efficiency, though TALENs retain unique advantages, such as PAM‐independent targeting and lower immunogenicity. Supported by powerful protein prediction tools like AlphaFold3, TALENs may experience renewed interest through structural optimization (e.g., high‐fidelity FokI variants), improved design platforms, and integration with CRISPR or small‐molecule systems. These advancements could expand their utility in genetic testing, precision medicine, and cell therapy, particularly in scenarios where CRISPR‐based tools are less suitable.

### 3.4 CRISPR/Cas System

**FIGURE 6:**
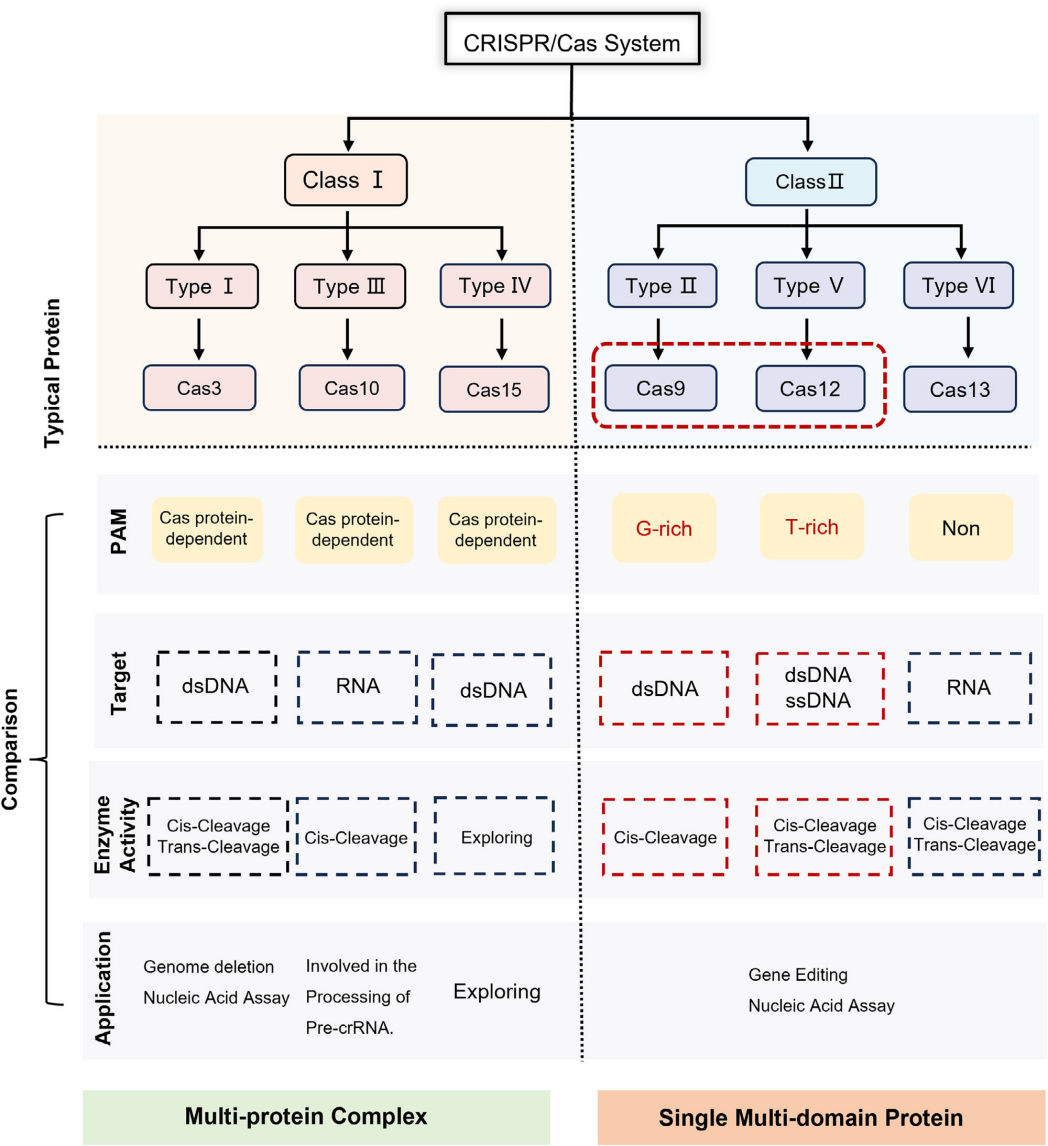
Classification of CRISPR/Cas system and representative proteins.

The CRISPR/Cas system is a powerful gene‐targeting tool with broad applications in gene editing, diagnostics, and disease treatment. Originally an adaptive immune mechanism in bacteria and archaea, it relies on Cas proteins and guide RNA (gRNA) to recognize and cleave foreign DNA. In 2012, Jennifer Doudna and colleagues [105] first demonstrated that the CRISPR/Cas system could be used to cleave any DNA sequence in vitro. Over the years, the CRISPR/Cas system has evolved into two major classes and six types, and their respective subtypes [106] (Figure 6). The CRISPR/Cas system is classified into two major classes: Class I (Types I, III, IV), which uses multiprotein complexes for defense, and Class II (Types II, V, VI), which relies on single Cas proteins and is more suitable for gene editing and diagnostics. Notably, representative members of the Class II system, such as Cas9, Cas12, and Cas13, are widely used in gene editing and disease treatment, where they guide RNA to recognize and cleave target DNA or RNA substrates.

Unlike ZFPs and TALENs, CRISPR/Cas is programmable via gRNA, enabling precise DNA targeting with a single Cas protein. Different Cas variants have distinct recognition mechanisms, making them versatile for various applications. Here, we mainly focus on the research progress and application potential of Cas9 and Cas12 proteins in dsDNA targeting. These two proteins were selected due to their broad application in gene editing and established technological foundations. Cas9, the earliest CRISPR protein developed, has made significant progress in research and achieved notable results in gene function studies, genetic imaging, and gene therapy. Cas12 has recently gained attention for its targeting properties and trans‐cleavage activity, particularly in genetic testing.

#### 3.4.1 Structural Basis of CRISPR/Cas9 and Its Applications in Gene Detection

**FIGURE 7:**
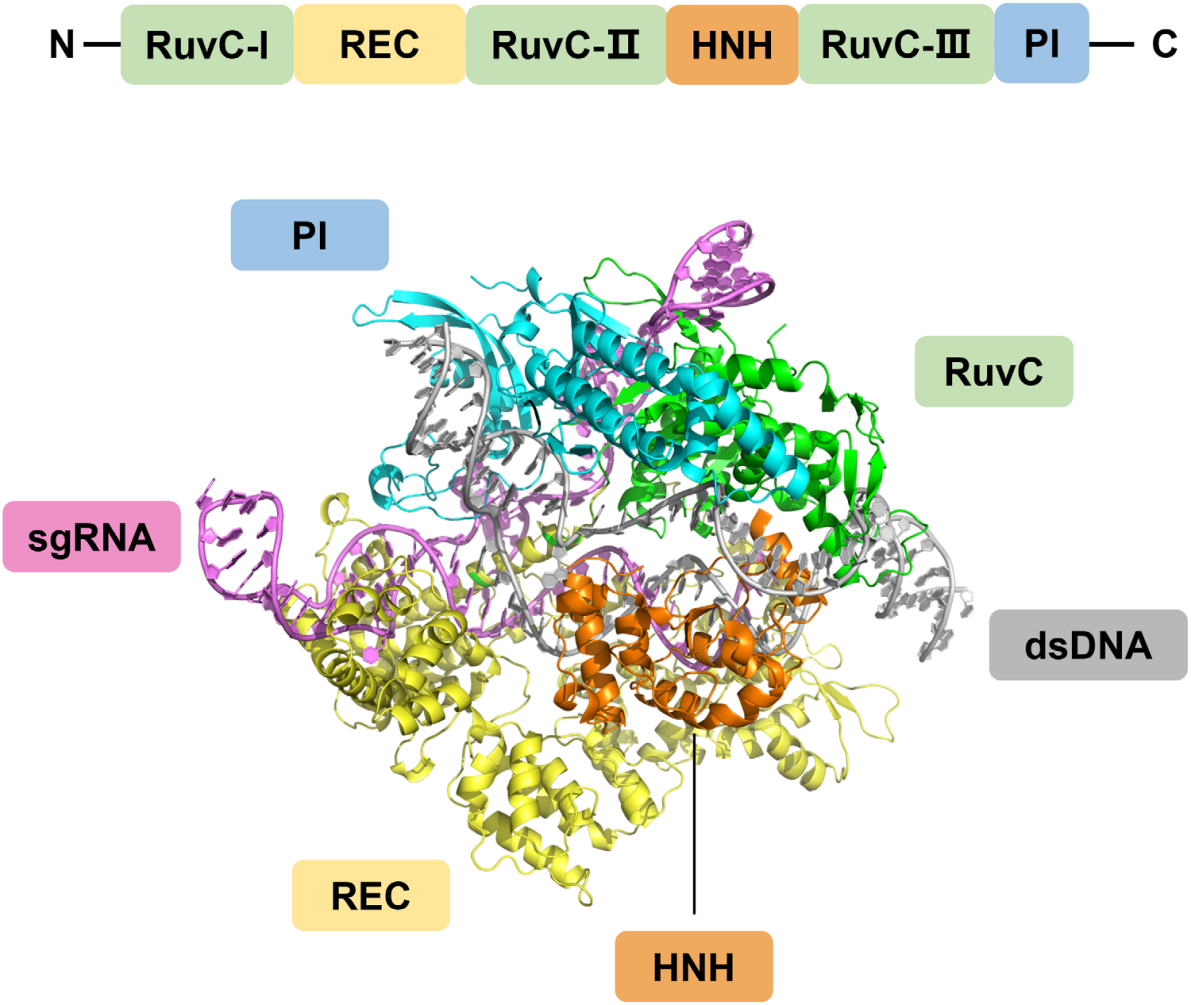
Crystal structures of spCas9 and gRNA, predicted by AlphaFold3 [88] and visualized using Pymol [107].

The targeting ability of the Cas9 is conferred by the gRNA bound to its REC domain. The gRNA comprises (CRISPR RNA) crRNA and trans‐activating CRISPR RNA (tracrRNA), which can form double‐stranded structures or combine into single‐guide RNA (sgRNA) [105]. The 20 bases at the 5' end of the sgRNA, known as the spacer, serve as a programmable sequence that determines Cas9's specificity, while the 3' end interacts with the REC domain. sgRNA interacts with dsDNA to form a three‐stranded dsDNA‐RNA hybrid structure (R‐loop) mediated by Cas9. The PI domain of Cas9 recognizes the protospacer adjacent motif (PAM) sequence (5'‐NGG‐3'). These two factors—the R‐loop formation and the PAM sequence recognition—work together to ensure the precise identification of the DNA target. spCas9, derived from *Streptococcus pyogenes*, is the most effective and widely used Cas9 nuclease (Figure 7). The REC domain of SpCas9 contains two arginine residues (Arg1333 and Arg1335) that form hydrogen bonds with the GG dinucleotide within the PAM sequence. Cas9's nuclease activity is mediated by the cooperative action of the RuvC and HNH domains. These domains cooperate to cleave the dsDNA substrate three nucleotides upstream of the PAM sequence, generating either blunt ends or DSBs.

**FIGURE 8:**
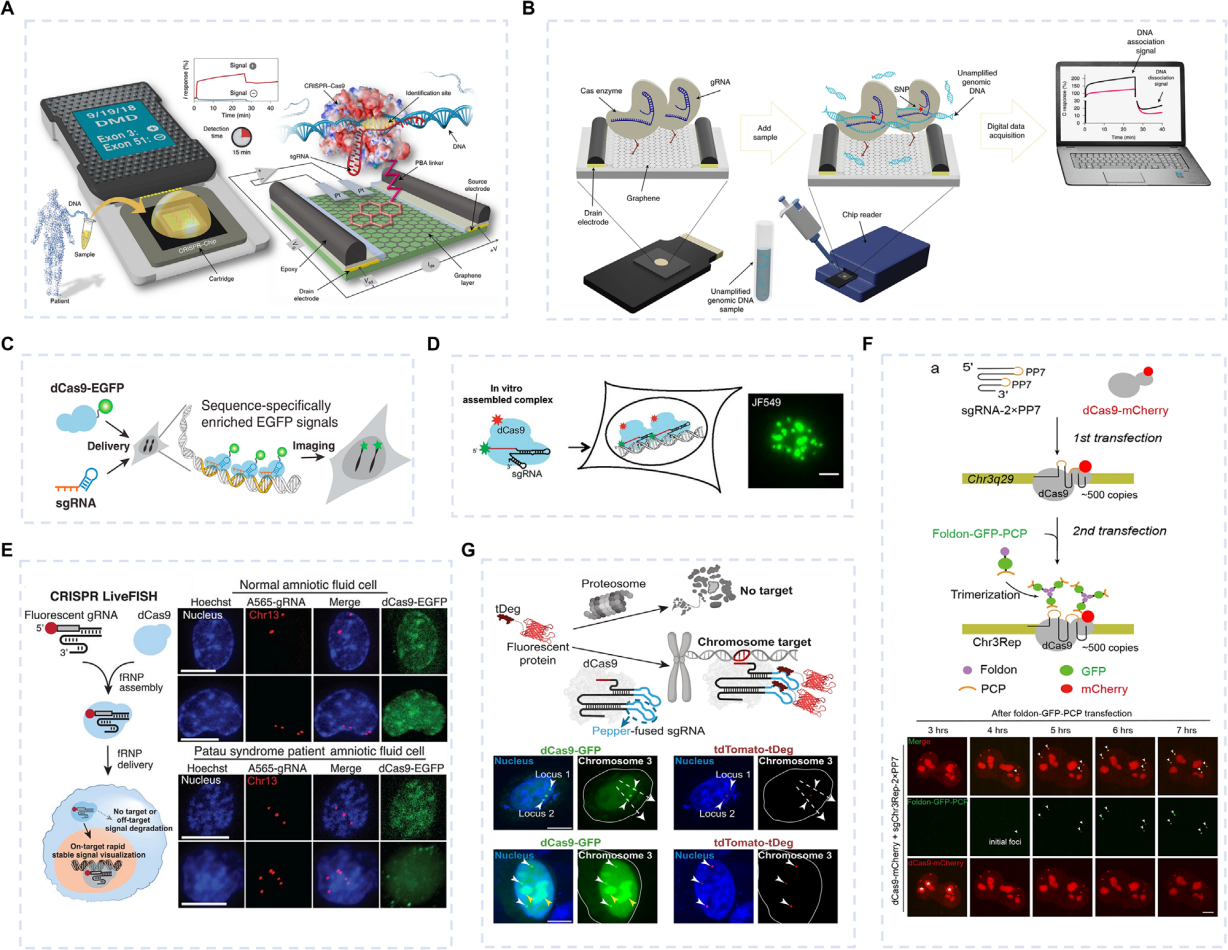
dsDNA detection by the CRISPR/Cas9 system. (A,B) CRISPR‐based graphene field‐effect transistor for ultrasensitive gene detection. Immobilization of (A) dCas9 [108] (Copyright 2019, Springer Nature) and (B) Cas9 [109] (Copyright 2021, Springer Nature) on graphene surface for gene detection in unamplified samples. (C–G) CRISPR‐based fluorescence in situ hybridization (CRISPR‐FISH) for chromosomal localization. (C) Fusion of Cas9 with GFP enables live‐cell imaging of chromosomal genes. Reproduced with permission [110]. Copyright 2013, Cell Press. (D) CASFISH uses Cas9 as a probe to label sequence‐specific genomic loci with fluorescence. Reproduced with permission [111]. Copyright  2015, National Academy of Sciences. (E) CRISPR‐LiveFISH detects DNA using fluorophore‐conjugated guide RNAs in association with Cas9. Reproduced with permission [112]. Copyright 2019, AAAS. (F) CRISPR FISHer facilitates sgRNA‐directed visualization of native non‐repetitive DNA loci in live cells. Reproduced with permission [113]. Copyright 2022, Springer Nature. (G) fCRISPR enables high‐sensitivity imaging of various genomic DNA in different human cell types, with an enhanced signal‐to‐noise ratio. Reproduced with permission [114]. Copyright 2024, Springer Nature.

Mutations at aspartic acid position 10 and histidine position 840 in the Cas9 protein result in the formation of nuclease‐deactivated Cas9 (dCas9). While dCas9 preserves the DNA‐targeting capability of Cas9 proteins, its nuclease activity is abolished, allowing it to serve as a powerful tool for gene expression regulation and genome labeling without inducing DSBs. This makes dCas9 adaptable to electrochemical analysis, especially graphene field effect transistor (GFET) sensors [115]. GFET sensors work with graphene channels and three electrodes (source, drain, and gate), and the carrier density is controlled by adjusting the gate voltage to change the electrical properties of graphene materials. After specific receptors are immobilized on the graphene surface, target molecules can be captured. The interaction between these molecules and graphene induces changes in its local electrical properties, which are then converted into detectable electrical signals, such as variations in source‐drain currents or shifts in the gate voltage. Due to their high sensitivity and fast response characteristics, GFET sensors offer significant advantages in the field of biochemical sensing. Hajian et al. [108] developed a GFET sensor called CRISPR‐Chip (Figure 8A), which indirectly immobilized dRNPs composed of dCas9 and sgRNA on the graphene surface of GFET via the amino group. This sensor captured the target gene and generated the corresponding electrical signals within 15 min, without the need for DNA amplification, achieving a detection limit of 1.7 fM. CRISPR‐Chip successfully identified the relevant gene mutations in Duchenne muscular dystrophy patients, showing the great potential of GFET in distinguishing mutated genes. Subsequently, Balderston et al. [109] displayed the SNP‐Chip by constructing a GFET sensor using Cas9 (Figure 8B). The sensor was able to accurately identify genomes associated with sickle cell anemia and amyotrophic lateral sclerosis in the genome, with a detection limit of 6.3 fM. The researchers finally utilized the SNP‐Chip to complete the testing of genome samples from SCD and ALS patients. Single‐nucleotide level specificity was demonstrated in detection. Notably, CRISPR‐GFET and Cas‐GFET systems detect signals through current changes and graphene transfer curve shifts, typically using manually defined thresholds. The recent integration of GFET signal processing with artificial neural networks (e.g., support vector machine [116], convolutional neural network [117]) has significantly improved detection robustness. Consequently, these machine learning models further optimize the performance of CRISPR/Cas‐based GFET systems.

The emergence of dCas9 has also ushered in a new era for in situ imaging of living cells, particularly addressing the challenge of imaging non‐repetitive sequences. Chen et al. [110] were the pioneers in developing dCas9 for CRISPR imaging (Figure 8C). They employed a method of tagging Cas proteins to deliver dRNPs fused with green fluorescent proteins into living cells, facilitating the localization of repetitive elements in telomeres and *MUC1* gene regions within living cells. This technique eliminates the denaturing step required in conventional FISH, offering a powerful and flexible approach for the dynamic visualization of arbitrary genomic sequences. Also, fluorescently labeling dCas9, CRISPR/Cas9‐mediated FISH (CASFISH) was proposed by Deng [111] (Figure 8D). A key innovation of this approach is the use of an sgRNA array, a systematically designed set of sgRNAs that tile specific genomic regions, thereby increasing dCas9 binding density. This strategy enables the visualization of non‐repetitive genomic loci with high precision and facilitates multicolor genome labeling through differentially labeled dRNPs, enhancing both imaging resolution and detection specificity. Subsequently, Wang et al. [112] changed the strategy of tagging proteins and proposed a robust and versatile method of CRISPR live‐cell FISH (LiveFISH) (Figure 8E), and they proposed fluorescent tagging of sgRNA for the first time to achieve accurate detection of chromosomal disorders in a wide range of cell types, including primary cells to detect chromosomal diseases. They selected two non‐repetitive genes, *PPP1R2* and *SPACA7*, as imaging loci, demonstrating this method's robustness. Based on the above work, the CRISPR genome imaging technology has been continuously optimized in terms of signal‐to‐noise ratio and sensitivity for imaging non‐repetitive sequences, and the current research is dedicated to tracking the dynamic behavior of chromosomes. Lyu et al. [113] constructed a CRISPR‐mediated fluorescence in situ amplifier system (CRISPR FISHer) that enables highly sensitive imaging of non‐repetitive DNA sequences in living cells (Figure 8F). By utilizing engineered sgRNA and protein trimerization‐mediated phase separation, this system achieved exponential assembly of fluorescent proteins at targeted sites, enhancing local brightness and signal‐to‐background ratios. CRISPR FISHer enabled real‐time visualization of chromosomal events, including DSBs, segregation, and reattachment, as well as tracking dynamic behaviors of extrachromosomal DNAs and invasive DNAs, revealing differences between chromosomal and extrachromosomal loci. Recently, Zhang et al. [114] also developed a fluorescent CRISPR (fCRISPR) (Figure 8G). fCRISPR included a fluorescent protein, which is degraded unless it binds to specific RNA hairpin structures. These hairpin structures are inserted into the sgRNA, forming a ternary complex of dCas9, sgRNA, and fluorescent protein, which enables fluorescent DNA imaging. They tracked chromosome dynamics and length through fCRISPR, as well as imaging *PPP1R2* and *SPACA7* genes to track DSBs and DNA repair.

#### 3.4.2 Structural Basis of CRISPR/Cas12 and Its Applications in Gene Detection

Cas12 is a family of single‐effector proteins in the CRISPR/Cas system, also referred to as Cpf1. This family includes different subtypes such as Cas12a, Cas12b, Cas12f, Cas12e, etc. These enzymes target both dsDNA and ssDNA, and each Cas12 enzyme recognizes a specific PAM sequence, which is essential for the target recognition and cleavage process. Within this family, Cas12a and Cas12b are well known for their diverse applications in genetic testing. However, they differ in structural domains, guide RNA, PAM sequence, and cleavage products, giving them distinct features in molecular diagnostics. Unlike Cas9, the WED structural domain of the Cas12a protein molecule has a ribonuclease site that permits autonomous processing of pre‐crRNA into mature crRNA without the need for additional tracrRNA or RNase III.

**FIGURE 9:**
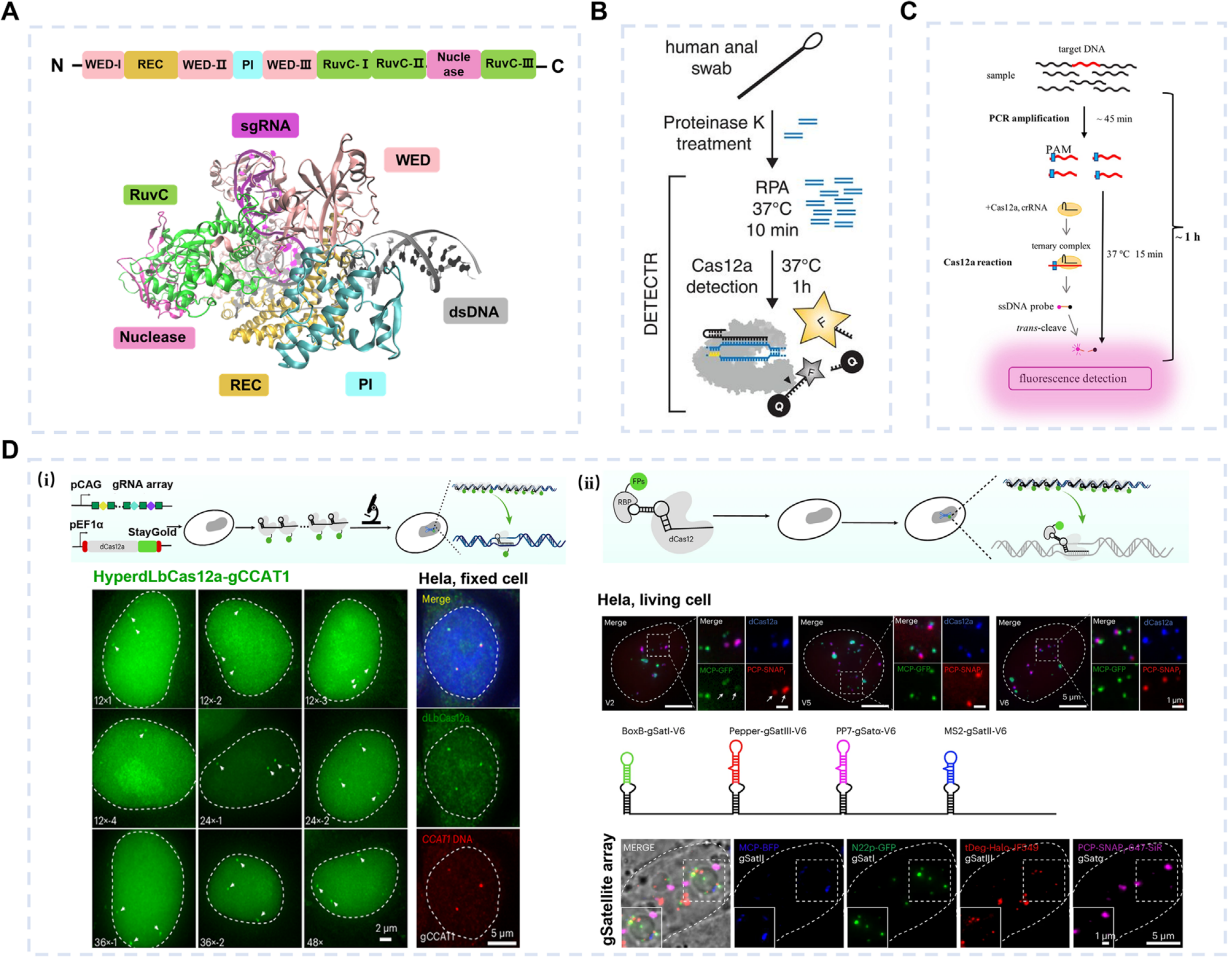
Cas12‐based dsDNA detection strategies. (A) The crystal structure of Cas12a was predicted by AlphaFold3 and visualized utilizing VMD [131]. (B) DETECTR combined Cas12a trans‐cleavage activity with RPA for dsDNA detection. Reproduced with permission [118]. Copyright 2018, AAAS. (C) HOLMES combined Cas12a trans‐cleavage activity with PCR reaction for dsDNA detection. Reproduced with permission [120]. Copyright 2018, Springer Nature. (D) HyperdLbCas12a for imaging non‐repetitive and multiple genomic loci in live cells. (i) CRISPR array visualizes non‐repetitive genomic loci through hyperdLbCas12a. (ii) Multiplex DNA imaging using a CRISPR array modified with a single RNA aptamer. Reproduced with permission [132]. Copyright 2024, Springer Nature.

After the Cas12a protein is directed to a specific target, the PI Domain recognizes binding to the target near the PAM sequence (5'‐TTTV‐3'), which is a key step for Cas12a to initiate R‐Loop formation (Figure 9A). The formation of the R‐loop activates the RuvC domain, enabling cleavage of dsDNA and generating sticky ends with a 3' overhang. Cas12b, lacking a ribonuclease active site, retains the crRNA and tracrRNA in its gRNA. Its PAM sequence (5'‐TTN‐3') differs from Cas12a, and cleavage produces a DNA fragment with a 5' sticky end and a 3' blunt end.

**TABLE 2:** Comprehensive comparisons of dsDNA targeting strategies.

	Hybridization probes	PNA and LNA	ZFPs	TALENs	CRISPR/Cas	pAgos	Lambda Exo‐pDNA
Reaction temperature	Annealing temperature	Annealing or room temperature	Room temperature	Room temperature	Room temperature or physiological temperature	Most Ago proteins require high temperatures for optimal function	Room temperature or physiological temperature
Off‐target	Low‐medium	Low‐medium	High	Medium‐high	High	N.A.	N.A.
Scalability level	High	Medium‐high	Low‐medium	Medium	Extremely high	Medium	Still in the research and having great scalability potential
Operational complexity	Easy, routine lab protocols	Moderate, requires expertise	Difficult, expert‐level skills	Challenging, time‐consuming	Relatively easy, widely accessible	Moderate, emerging technology	Easy, minimal expertise required
Influence factors on specificity	C/G content, probe length annealing temperature	Strong affinity, mismatch tolerance	Interaction between motifs, space's length	Spacer's length, T‐base initiation	PAM sequence, mismatch distance, design of gRNA	Temperature dependence, guide sequence preference	pDNA secondary structure, buffer composition, λ exo concentration
Reagent cost	Low (DNA probes, fluorophores, PCR reagents)	Medium‐high (commercial synthesis)	High (protein expression, purification, validation)	High (TALE protein assembly, cloning reagents)	Low‐medium (sgRNA synthesis, Cas protein, delivery reagents)	Medium (Ago protein, limited suppliers)	Low (λ Exo, pDNA, basic reagents)
Main limitations	Limited flexibility	High‐cost, complex synthesis	Complex customization, low throughput	Labor‐intensive, low delivery efficiency	PAM dependency	Immature, lacks commercial support	Dependent on downstream methods, limited standalone use
Advantages	Mature technology, low cost, high throughput	High specificity and affinity	Precise targeting	Modular design	High flexibility, mature high‐throughput	No PAM restriction, simple operation	Low‐cost, easy to design
Main applications	PCR primer, in situ imaging molecular diagnosis	SNV detection	Gene editing, molecular diagnosis	Gene editing	Multiplex gene editing and diagnostics	Molecular diagnosis, in situ imaging	Molecular diagnosis, in situ imaging

Cas9 is primarily used in applications related to its main activity, which is the direct cleavage of target DNA, or the binding activity of dCas9. In addition to their main activity, both Cas12a and Cas12b possess trans‐cleavage activity, enabling them to degrade ssDNA or ssRNA in the system nonspecifically, and they can maintain their enzymatic activity continuously. Doudna's laboratory was the first to disclose the trans‐cleavage activity of Cas12 and pioneered the first Cas12‐based dsDNA detection method— DNA endonuclease targeted CRISPR trans reporter (DETECTR) [118] (Figure 9B). In this method, the Cas12a enzyme derived from *Lachnospiraceae* bacterias (LbCas12a) is guided by crRNA to the dsDNA target amplified by RPA. Once successfully located, Cas12a activates its trans‐cleavage activity to cleave ssDNA reporters labeled with a fluorescent and a quencher group, generating a detectable fluorescent signal, with a detection limit at the amol level. This trans‐cleavage activity can also be combined with various pre‐amplification techniques, such as PCR‐coupled one‐hour low‐cost multipurpose highly efficient system (HOLMES) [119, 120, 121, 122, 123] (Figure 9C), as well as other reaction systems like loop‐mediated isothermal amplification (LAMP) [124, 125, 126, 127], and RPA [128, 129], further enhancing the sensitivity of the Cas12 system and expanding its application scenarios, as shown in Table 3. Similar to the Cas12‐based reporting system, Cas13‐based nucleic acid detection technologies target ssRNA. The specific high‐sensitivity enzymatic reporter unlocking (SHERLOCK) developed by Zhang Feng's team [130] played a crucial role in the fight against SARS‐CoV‐2. Given the widespread discussion of SHERLOCK in other RNA‐targeting literature, this review will not delve into its details. A comprehensive comparison of the characteristics of Cas proteins with other targeting technologies is presented in Table 2.

**TABLE 3:** Applications of dsDNA targeting technologies in diagnosis.

Targeting technologies	Diseases/Pathogens	Gene	Source of simple	Amplification	Signal output platform	LoD/VAF	References
Denaturation‐enabled hybridization	Multiple myeloma	*TP53, RB1, FGFR3/IGH*, etc.	Bone marrow aspirate	—	FISH	—	[189]
	Gastroesophageal adenocarcinoma (GEA)	*HER2, RAI1*	GEA tissues	—	FISH	—	[190]
	Salivary duct carcinoma (SDC)	*ERBB2*	SDC tissues	—	FISH	—	[191]
	Acute myeloid leukemia	*AML1/ETO*	leukocytes	—	FISH	—	[192]
Strand‐assisted hybridization	Cancer	ctDNA (*KRAS, BRAF*)	Human serum sample	—	Nano electrodes	1 fg µL^−1^	[54]
	Cancer	ctDNA (*BRAF*)	Human serum sample	—	Fluorometer	0.85 nM	[193]
	Soil‐transmitted helminths	β‐tubulin	Human feces	PCR	Fluorometer	1 aM	[55]
	Non‐small cell lung cancer	*EGFR L858R*, *19del* and *T790M*	Human plasma, pleuroperitoneal fluid, tumor tissue	PCR	Fluorometer	0.01%	[56]
Locked nucleic acid	Cancer	*EGFR T790M*	Mimic human serum sample	RRA, PCR	Fluorometer	0.007%	[60]
	—	*DYZ‐1*	Bos taurus	—	FISH	—	[72]
Peptide nucleic acid	Hepatitis A virus (HAV) Human immunodeficiency virus (HIV) Hepatitis B virus (HBV)	—	Synthetic target sequence	—	Fluorometer	260 pM	[57]
	Hepatitis C Virus (HCV)	—	Plasmid pBKC84	—	Electrochemical Assay	9.5 pg mL^−1^	[194]
ZFPs	*Staphylococcus aureus*	*SEB*	Synthetic target sequence	—	Chemiluminescence	50 fM	[92]
	*Escherichia coli* O157:H7	*STX2*	Synthetic target sequence	—	Absorption spectrometry	10 fM	[94]
	Infectious salmon anemia	Genome fragments	*Piscirickettsia salmonis*	RPA	Electrochemical Assay	300 copies/13.2µL	[90]
	Legionella pneumophila *Escherichia coli* O157	Genome fragments	Synthetic target sequence	PCR	Fluorometer	10 copies	[195]
TALENs	The primary application is in the field of gene editing.						
CRISPR/Cas	Acute myeloid leukemia	*NMP1*	Human blood	ERA	Lateral flow strips, portable fluorometer	0.01%	[196]
	Sickle cell disease, Amyotrophic lateral sclerosis	*HBB* (SCD) *ALS* (SOD1)	Human genome DNA	—	GFET	1.7 fM	[109]
	Non‐small cell lung cancer	*EGFR*	Human oral swab, serum sample	RPA	Fluorometer	16.8 aM	[197]
	Monkeypox	—	Human rash fluid, oral swab, saliva, urine	RPA	Fluorometer	0.5 copies/mL	[198]
	Human papillomavirus (HPV)	—	Human cervicovaginal swab samples	RPA	Fluorometer	40 copies/mL	[199]
	Human papillomavirus (HPV)	—	Human cervical secretion	—	Nano electrodes	1 × 10^−18^ M	[200]
Agos	*Enterocytozoon hepatopenaei*	*SWP*	Shrimp	RPA	Fluorometer	1 copy/µL	[201]
	*Salmonella typhimurium*	*invA*	Milk, eggs	LAMP	Lateral flow assay	1 CFU/mL	[202]
	Carbapenemase‐producing *Klebsiella pneumoniae*	*KPC*, *IMP*, *VIM*, *NDM*, *OXA‐48*	Urine, blood, micropigskin, and rectal swab samples	—	Fluorometer	1.87 fM (KPC) 178 aM (IMP) 529 aM (VIM) 120 aM (NDM) 144 aM (OXA‐48)	[174]
	*S. typhimurium*, *L. monoListeria monocytogenescytogenes*	Genome fragments	Poultry and pork samples	RPA	Microdroplet area and Fluorometer	21 CFU/mL	[203]
	HPV	—	Anal swabs	PCR	Fluorometer	Femtomolar level	[204]
λ exo‐pDNA system	*Enterococcus faecalis*	*ddl*	Synthetic target sequence	PCR	Fluorometer	0.1 nM	[186]
	*S. aureus*	*nuc*					
	*K. pneumoniae*	*mdh*					
	*Acinetobacter baumannii*	*gltA*					
	*Pseudomonas aeruginosa*	*oprL*					
	*Escherichia coli*	*ampC*					
	Human breast cancer	*KRAS*	MCF‐7 and MDA‐MB‐231 cell line	PCR	Fluorometer	—	
	—	*MUC4* and telomeres	A549 cell line	—	FISH	—	

dCas12 can also be used for cellular imaging. Compared to dCas9, dCas12's T‐rich PAM sequence enables it to target a broader range of genomic regions, making it especially effective for imaging non‐repetitive DNA regions. Yang et al. [132] recently demonstrated that HyperdLbCas12a outperforms other dCas proteins in live‐cell imaging of non‐repetitive DNA and developed the CRISPRdelight system (Figure 9D). Their work focused on the relationship between gene expression and nuclear localization by imaging the *CCAT1* and *HSP* loci. By engineering CRISPR arrays, they achieved dynamic imaging of non‐repetitive and multiple genomic loci in live cells. Additionally, by combining RNA aptamers, they could use a single array for multiplex imaging of four types of satellite DNA loci, revealing their spatial proximity to nucleolus‐associated domains.

#### 3.4.3 Progress in Gene Editing With CRISPR/Cas Systems

**FIGURE 10:**
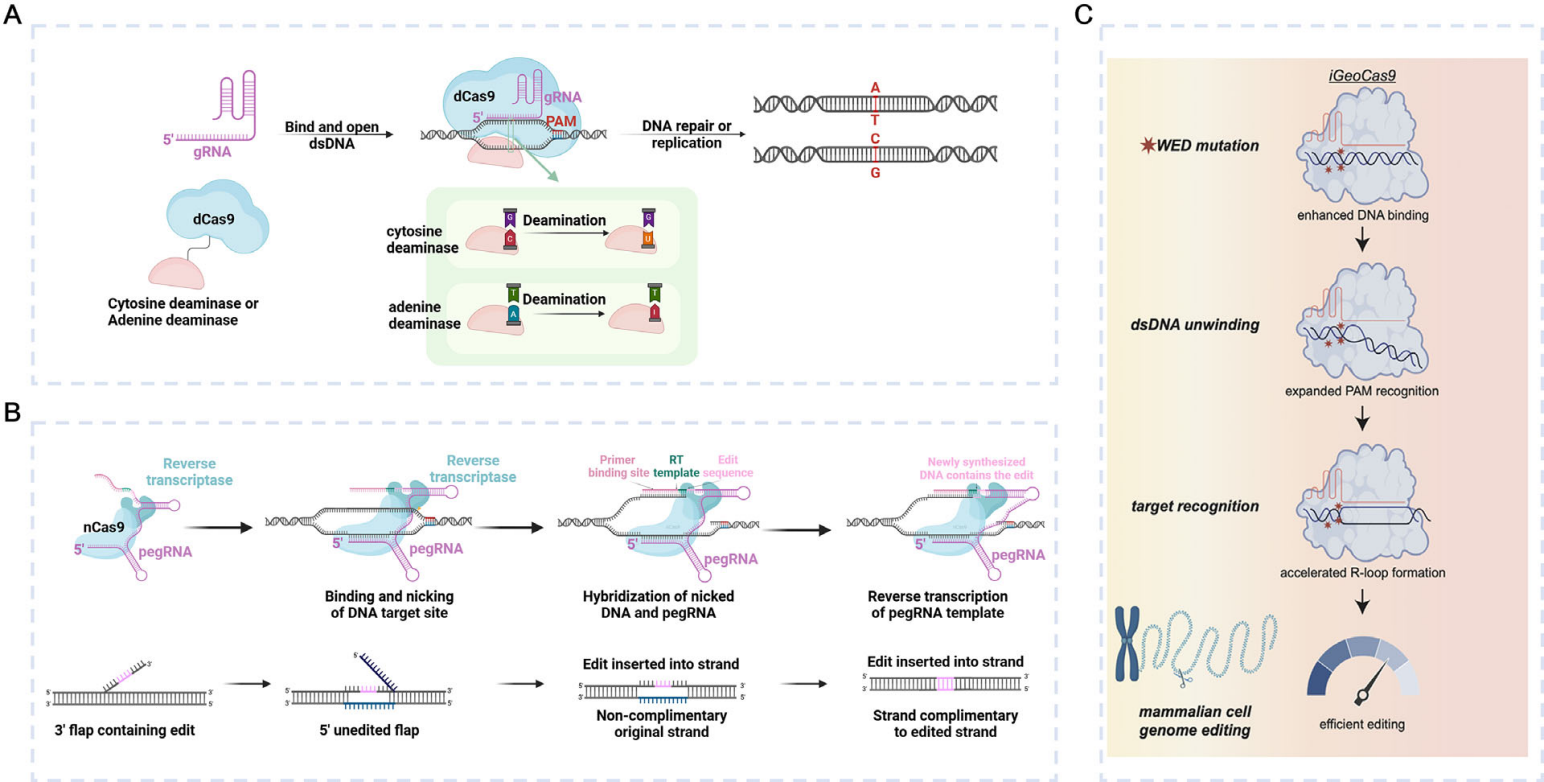
CRISPR/Cas system for gene editing. (A) Base editors enable precise base conversion by fusing dCas9 with functionalized enzymes. (B) Prime editing enables more diversified and directed genome DNA modifications by fusing dCas9 with reverse transcriptase. (C) iGeoCas9 accelerates the unwinding speed of dsDNA and exhibits enhanced editing efficiency. Reproduced with permission [133]. Copyright 2024, Cell Press.

The CRISPR/Cas system is currently the leading tool for gene editing [19]. The early CRISPR/Cas system for gene editing was confined to generating gene knockouts [134]. In 2016, David R. Liu's team developed the base editors (BEs) [135, 136, 137, 138, 139, 140], which fused dCas9 with functional enzymes like cytosine deaminase and adenine deaminase, enabling precise base transitions (C‐T, G‐A, A‐G, T‐C) without DSBs (Figure 10A). In 2019, Liu's team introduced the prime editors (PEs) [141, 142] (Figure 10B), which combined Cas9 with reverse transcriptase and utilized prime editing guide RNA (pegRNA) for efficient execution of 12 base conversions, as well as insertions (up to 44 bp) and deletions (up to 80 bp). Furthermore, improving editing efficiency and minimizing off‐target effects are key challenges in gene editing. To address these, Jennifer Doudna's team recently exhibited iGeoCas9 [133, 143] (Figure 10C), a thermally stable Cas9 variant. This variant has a mutated WED domain that enhances binding affinity to dsDNA and accelerates DNA unwinding, achieving 16% to 37% editing efficiency in mouse liver and lung cells [143].

The size of Cas proteins also affects delivery and editing efficiency, with smaller proteins enhancing cell entry and editing accuracy. Recent developments have introduced miniaturized Cas12 proteins [144, 145, 146, 147, 148, 149, 150], which may be utilized in diversity editing applications in the future.

#### 3.4.4 PAM Sequences and Off‐Target Effects in CRISPR/Cas Systems

The presence of PAM limits the targetable loci of the CRISPR/Cas system. For example, SpCas9 recognizes the NGG PAM, restricting its ability to target AT‐rich regions. Cas12 recognizes T‐rich PAMs, which limits their efficiency in targeting CG‐rich loci. Strategies such as using suboptimal PAMs [151, 152, 153] (e.g., NAG for SpCas9), engineering Cas proteins, and using high‐fidelity Cas proteins (e.g., eSpCas9 [154, 155], SpCas9‐HF1 [156], and LZ3 Cas9 [157, 158]) have been developed to expand the target range [159, 160].

The natural CRISPR/Cas9 system allows some degree of mismatch between the gRNA and target DNA, which is crucial for bacterial defense but can lead to off‐target effects in gene editing and detection. Off‐target effects correlate with mismatch distance from the PAM, with mismatches near the seed region having a greater impact [161], while distal mismatches may potentially reactivate Cas9 [162]. To reduce off‐target effects, optimizing the sgRNA's secondary structure [163, 164, 165, 166], and engineering proteins [154, 155, 156, 157, 158, 160] can enhance Cas9 specificity. The Cas12 family, with lower off‐target effects than Cas9, offers better accuracy, and optimization strategies continue to improve precision. Additionally, AI tools like AlphaFold3 [88], OpenCRISPR‐1 [167], EVO [168] are advancing CRISPR optimization by predicting off‐targets, refining gRNA designs, and modeling protein‐DNA interactions, significantly improving accuracy and efficiency.

Despite challenges with PAM limitations and off‐target effects, the CRISPR/Cas system holds vast potential for dsDNA targeting research and applications. Advances in CRISPR/Cas optimization and AI‐driven predictive models have significantly enhanced its precision and efficiency. Scientists are also exploring natural RNP systems similar to CRISPR/Cas in the meantime.

### 3.5 Argonaute Protein System

Argonaute proteins (Agos) are RNP gene‐targeting systems similar to the CRISPR/Cas systems. The Agos are widely distributed in the biological world, playing a crucial role in regulating gene expression and defending against foreign nucleic acid invasions. According to their origin, Agos can be classified into eukaryotic Agos (eAgos) and prokaryotic Agos (pAgos). In nature, eAgos are involved in RNA interference, facilitating the formation of RNA‐induced silencing complex, which cleaves specific mRNAs and inhibits their translation process. eAgos are relatively well conserved and will not be repeated in this review. In contrast, as a bacterial defense mechanism against foreign nucleic acid invasion, pAgo exhibits natural biological functions comparable to the CRISPR/Cas system. The core components of the pAgo system consist of an effector protein and a programmable guide nucleic acid, typically ssDNA or RNA phosphorylated or hydroxylated at the 5' end, ranging from 19 to 25 nucleotides in length. Unlike the CRISPR/Cas system, pAgo proteins do not require a PAM sequence, making them potentially more broad and specific molecular tools. In this review, we will examine the mechanism and applications of pAgo in gene recognition.

**FIGURE 11:**
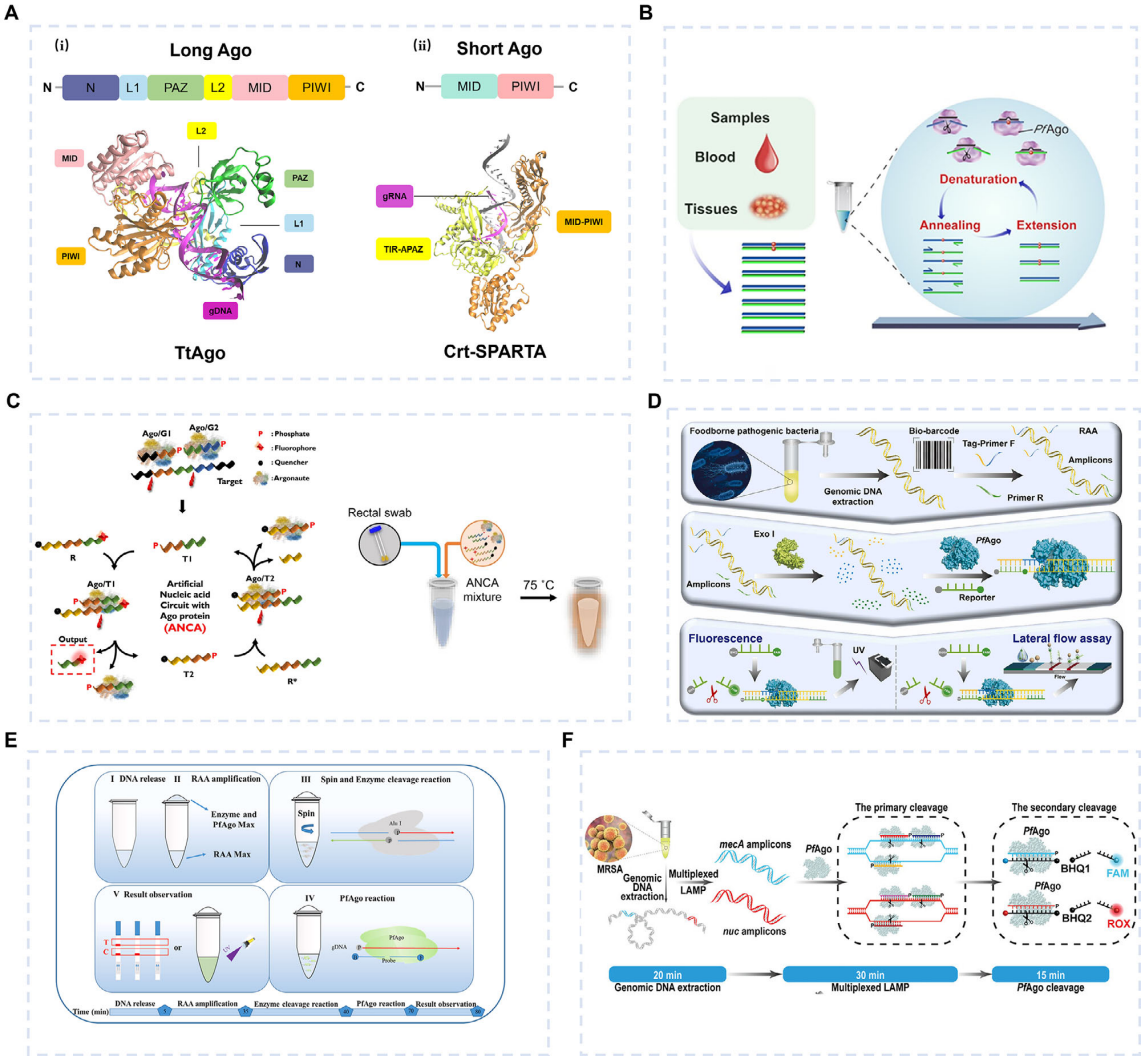
dsDNA detection techniques based on Ago proteins. (A) Crystal structures of (i) long pAgos and (ii) short pAgos, predicted by AlphaFold3 [88] and visualized using VMD [131]. (B) The A‐Star system, an integrated single‐tube PCR system with pfAgo, can eliminate wild‐type, thereby enriching mutant targets. Reproduced with permission [173]. Copyright 2021, Oxford University Press. (C) The ANCA system uses pfAgo to design artificial nucleic acid circuits for one‐step isothermal detection of antibiotic‐resistant bacteria. Reproduced with permission [174]. Copyright 2023, Springer Nature. (D) pfAgo reaction to introduce tagged sequences and incorporate RAA for LFA detection. Reproduced with permission [175]. Copyright 2024, Elsevier. (E) REPD system combining RAA amplification, restriction endonuclease digestion, and PfAgo reaction to achieve one‐pot detection of ASFV. Reproduced with permission [176]. Copyright 2024, Elsevier. (F) STAR system combines PfAgo reaction and LAMP to achieve detection of *mecA* and *nuc* genes of MRSA. Reproduced with permission [177]. Copyright 2024, Wiley.

pAgo can be categorized into long pAgo and short Ago based on their molecular weights. For long pAgos, the N‐terminus consists of the N‐terminal domain, L1 linker, and PIWI‐Argonaute‐Zwille (PAZ) domain, and the C‐terminus comprises the MID domain, L2 linker, and PIWI domain. The N‐terminal domain's role remains less well‐defined but appears to contribute to several processes. It is thought to modulate target recognition, assist in unwinding the guide‐target duplex, and promote target DNA binding by inducing conformational changes in the protein. The L1 linker, a flexible region, connects the N‐terminal domain to the PAZ domain, facilitating structural adaptability. The PAZ domain primarily anchors the 3' end of the guide strand, securing it during the targeting process. In contrast, the MID domain binds the 5' phosphate of the guide strand, playing a critical role in guide loading. By collaborating with the PIWI domain, the MID domain ensures precise positioning of the guide strand at the active site. Similar to the L1 linker, the L2 linker provides structural flexibility, connecting the MID and PIWI domains. This flexibility allows the PIWI domain to adjust its orientation for effective target binding or cleavage. The PIWI domain serves as the catalytic core of Ago proteins, exhibiting RNase H‐like activity responsible for cleaving target DNA. It contains a conserved catalytic triad or tetrad—typically consisting of Asp, Glu, and His residues—that hydrolyzes the phosphodiester bond in the target DNA. The presence and functionality of this catalytic site determine whether an Ago protein acts as a “slicer” with cleavage activity or a “non‐slicer” lacking such capability. Together, these two termini form a bilobed structure (Figure 11A(i)). The bilobed structure exposes the seed region of the guide nucleic acid, offering a high‐affinity surface for target DNA binding. For short pAgos, the C‐terminus also contains MID and PIWI domains, while the N‐terminal lobe is absent [169, 170, 171]. Therefore, short pAgo has to recruit some additional proteins, such as SIR2, TIR, and APAZ, to compensate for the N‐terminal function [170] (Figure 11A(ii)). By comparison, long pAgos are widely studied due to their broad applications, particularly pfAgo from *Pyrococcus furiosus* [172] and TtAgo from *Thermus thermophilus* (Figure 11A(i)). These two Agos have become research focal points because of their unique thermal stability.

Agos have been attempted to be developed as gene editing tools, but their applications have been limited due to suboptimal results. Nowadays, Agos are predominantly utilized in gene detection systems [178, 179]. For example, Liu et al. [173] developed a single‐tube, multiplex PCR‐based system called A‐Star, incorporating PfAgo for detecting rare mutations (Figure 11B). This system enabled PfAgo to selectively cleave DNA substrate with single‐nucleotide resolution at 94°C, thereby efficiently amplifying rare mutant DNA during the PCR process. Consequently, the A‐Star system facilitated the detection and quantification of rare mutations at a frequency as low as 0.01%, achieving a ≥5500‐fold increase in efficiency. Jang et al. [174] reported an artificial nucleic acid circuit with Ago protein (ANCA) for one‐step, amplification‐free, and isothermal DNA detection of antibiotic‐resistant bacteria genes (Figure 11C). To function synergistically, the ANCA established a positive feedback circuit by designing four signal processors (G1, G2, R, and R*). By utilizing the cross‐catalytic cleavage ability of the TtAgo reaction, the ANCA derived a continuous autocatalytic reaction that results in signal amplification. Eliminating the need for a DNA extraction and amplification step, the technique can detect carbapenemase‐producing *Klebsiella pneumoniae* in human urine, blood, and rectal swab specimens of infected patients with a LOD of 1.87 fM, demonstrating 100% sensitivity and specificity. Li et al. [175] exhibited an isothermal amplification method based on recombinase‐aided amplification (RAA) and lateral flow assay (LFA) (Figure 11D), incorporating an Argonaute‐mediated bio‐barcoding bioassay for the rapid point‐of‐care testing of *Staphylococcus aureus*. The forward primer was barcoded with a tag sequence in the amplification strategy. After the amplification reaction, the tag sequence was barcoded to the amplification product. Then, Exo I was added to digest the excess primers. Subsequently, the digested amplification product was used for triggering the PfAgo reaction, resulting in cleavage of the F‐Q reporter gene. The final signal readout was achieved through fluorescent signal detection or LFA, with a 1 CFU/mL detection limit. Zhao et al. [176] showed REPD (Figure 11E), a one‐pot method combining RAA reaction and restriction endonuclease‐assisted PfAgo detection. The one‐pot REPD could detect a single copy of African Swine Fever Virus (ASFV) nucleic acids with a detection limit of 1 copy/mL, exhibiting no cross‐reactivity with other pathogens. Kou et al. [177] developed a portable pfAgo‐centered biosensor (STAR) (Figure 11F) for the detection of methicillin‐resistant *Staphylococcus aureus* (*MRSA*). Specifically, the species‐specific mecA and nuc genes are amplified isothermally using LAMP. This is followed by Argonaute‐based detection, which uses its secondary cleavage reaction to convert target nucleic acid signals into fluorescent signals. The detection limit is 10 CFU/mL. Certainly, in addition to thermophilic Ago proteins such as pfAgo and TtAgo, there are a series of other Argonaute proteins, such as CbAgo [180], CdAgo [181], and salt‐tolerant NgAgo [182], etc. These proteins, each with its unique characteristics, are applied across various contexts based on their specific attributes.

Research on Agos remains limited compared to CRISPR/Cas systems. Agos offer advantages such as PAM‐independent targeting and high specificity due to seed region mismatch sensitivity. However, mature Ago proteins (e.g., pfAgo, TtAgo, NgAgo) often require harsh conditions (e.g., high temperature or osmotic pressure), limiting their practical use. Efforts to engineer Agos aim to enhance stability and activity under mild conditions while retaining functionality in extreme environments, expanding their utility in diagnostics and therapeutics. Supported by molecular prediction tools, the exploration of novel Agos with diverse structural and functional properties could unlock broader applications in the future.

### 3.6 Lambda Exonuclease‐pDNA System

**FIGURE 12:**
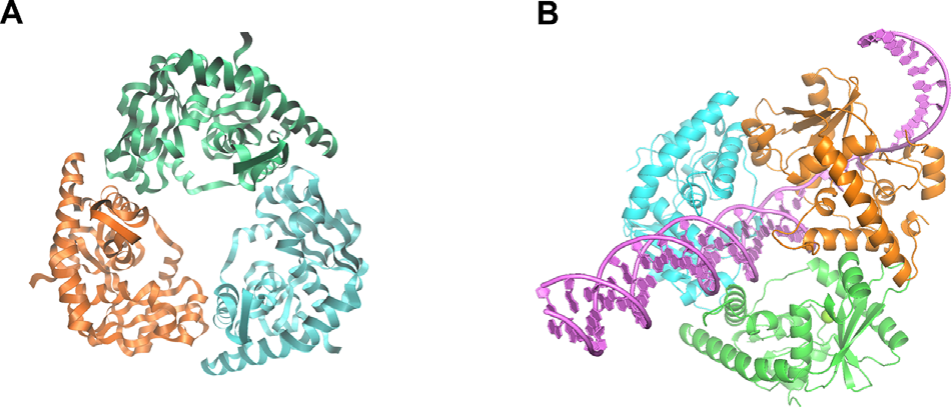
Crystal structures of Lambda Exonuclease predicted by AlphaFold3 [88]. (A) The homologous trimeric structure of λ Exo, visualized using VMD [131]. (B) The process of λ Exo cleaving 5'‐phosphorylated dsDNA, visualized using Pymol [107].

**FIGURE 13:**
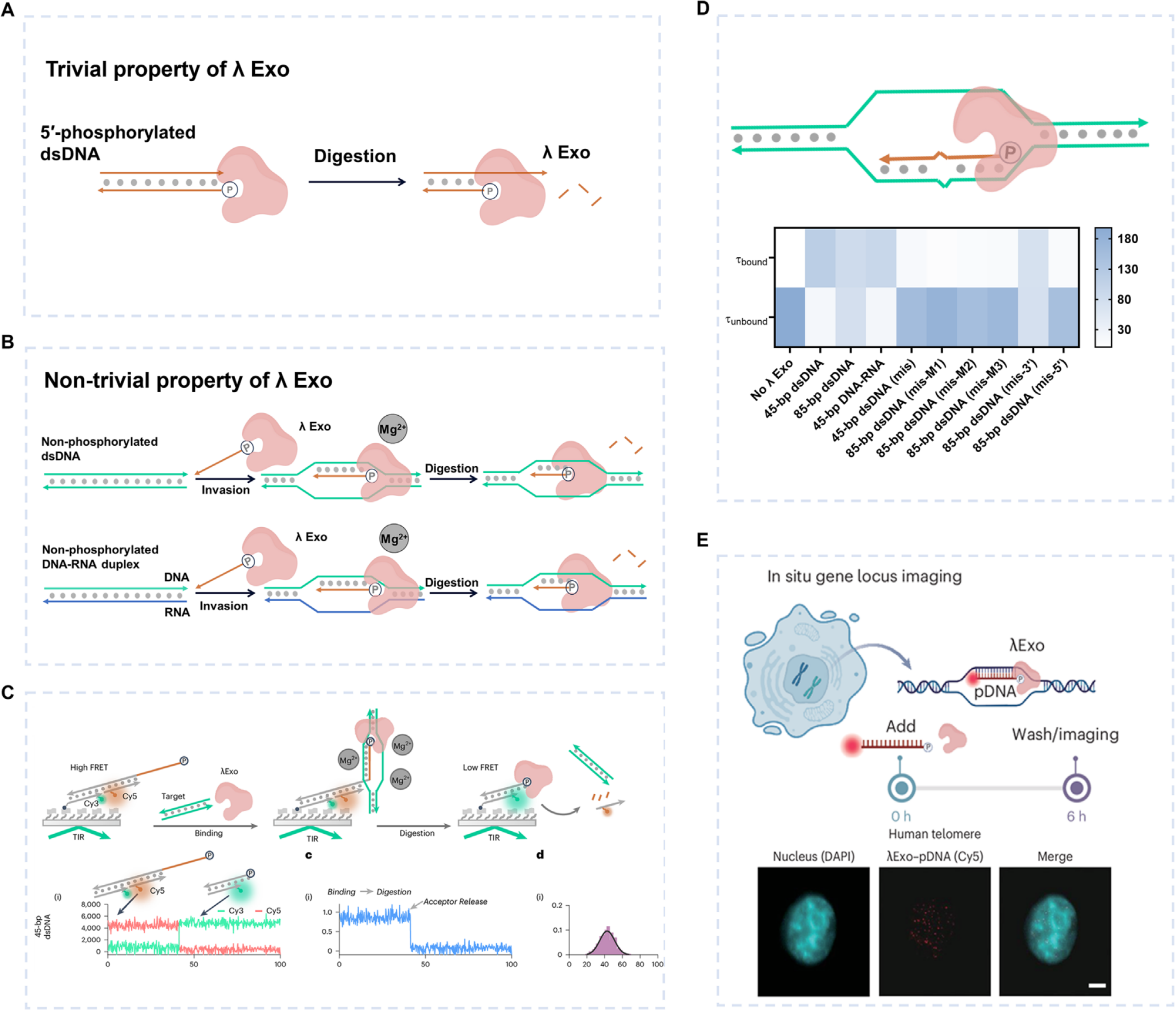
dsDNA detection based on λ Exo‐pDNA system. (A) The trivial property of λ Exo, selective degradation of the 5′‐phosphorylated strand of dsDNA. (B) The non‐trivial property of λ Exo, selective degradation of single‐stranded pDNA inserted into dsDNA or DNA/RNA hybrids. (C) Characterization of λ Exo‐pDNA cleavage activity using single‐molecule fluorescence experiments. (D) The enzymatic activity of λ Exo is sensitive to mismatch. (E) In situ imaging of cellular genes based on the λ Exo‐pDNA system. (C–E) Reproduced with permission [186]. Copyright 2024, Springer Nature.

Lambda Exonuclease (λ Exo), derived from bacteriophages, is a highly specific 5'‐3' nucleic acid exonuclease that selectively degrades linear dsDNA phosphorylated at the 5' end (Figures 12B and 13A) [183, 184]. The λ Exo protein, with a molecular weight of 25 kDa, is significantly smaller than both Cas proteins (∼100 kDa) and long Argonaute proteins (∼78–98 kDa). This unique miniaturization advantage makes it particularly suitable for intracellular applications in living cells. Its crystal structure (Figure 12A) reveals a distinctive homotrimeric arrangement, forming a funnel‐shaped central channel. The entrance of this channel has a diameter of approximately 30 Å, which is large enough to accommodate dsDNA; while the exit narrows to around 15 Å, permitting only ssDNA to pass through [185]. This intricate geometric design provides a unique platform for the specific degradation of DNA: 5′‐phosphorylated dsDNA smoothly enters the wide opening of the funnel, after catalysis by the exonuclease, single‐stranded DNA (ssDNA) is released through the narrow exit. This process requires Mg^2+^ as a cofactor but does not rely on ATP or GTP, a well‐known feature of λ Exo activity. Recently, Su's team [186] made a groundbreaking discovery that 5′‐phosphorylated ssDNA (pDNA) can insert into dsDNA targets or DNA/RNA hybrids containing complementary regions to the pDNA. Subsequently, λ Exo degrades this pDNA from the 5′ end (Figure 13B). This process is entirely PAM‐independent and can occur at room or physiological temperatures, revealing a previously unknown function of λ Exo.

In their work, single‐molecule fluorescence resonance energy transfer (smFRET) assays were performed using total internal reflection fluorescence microscopy to investigate λ Exo‐mediated dsDNA interrogation [187, 188]. Unlike ensemble FRET, which monitors the fluorescence signal of the system as a whole, smFRET can accurately measure the fluorescence signal of individual molecules on the scale of 1–10 nm. It was found that divalent cations were essential. In the presence of Ca^2^^+^, pDNA and λ Exo remain bound to the targets, resembling the behavior of dCas9. Whereas in the presence of Mg^2^^+^, pDNA is rapidly degraded by λ Exo (Figure 13C). Interestingly, when the λ Exo‐pDNA system interacted with DNA‐RNA complexes, λ Exo could only digest pDNA complementary to DNA, not RNA. In addition, the team observed that the enzymatic activity of λ Exo was sensitive to mismatches (Figure 13D). The binding efficiency of pDNA was significantly reduced when the dsDNA target was 45 bp in length and contained mismatches. For the slightly longer 85 bp dsDNA, mismatches located at the middle and 5′ ends significantly inhibited pDNA binding, whereas mismatches at the 3′ end had little impact. Electrophoretic experiments further confirmed that mismatches significantly hindered the formation of the ternary complex when λ Exo and pDNA bound the target dsDNA.

Based on the novel properties of λ Exo, they tested gene fragments from six highly virulent and drug‐resistant bacteria. No cross‐reactivity was observed, demonstrating the high specificity of the λ Exo‐pDNA system. The researchers then designed and conducted further experiments, including in situ imaging of the *MUC4* gene in A549 human cells (Figure 13E), a logic circuit experiment based on dsDNA response, and integration with HCR. These experiments demonstrated the significant potential of the λ Exo‐pDNA system as a novel probe in molecular diagnostics and gene imaging applications. The advantages, limitations, and key features of λ Exo‐pDNA system are summarized in Table 2.

## 4 Other Cutting‐Edge dsDNA Targeting Technologies

With the continuous advancement of cell biology and protein research, many molecular recognition systems have been discovered and studied. A common feature of these systems is that they all are composed of guide nucleic acids and effector proteins. Similar to the previously discussed CRISPR/Cas, Argonaute proteins, and λ Exo‐pDNA system, their guide nucleic acids also exhibit strong programmability. By designing and adjusting the guider's sequences, these systems can precisely recognize and regulate specific genes.

In exploring the origin of the CRISPR‐Cas system, researchers discovered the value inherent in insertion sequences, which are fundamental mobile genetic elements. Particularly notable is the widely distributed *IS200/IS605* gene family in nature, which encodes IscB, IsrB, TnpB [205], and various transposon proteins. These proteins are also RNA‐guided DNA nucleases, referred to as ωRNA‐guided nucleases, and are collectively named obligate mobile element‐guided activity systems (OMEGA). Subsequent studies revealed a clear evolutionary relationship between OMEGA and the CRISPR/Cas system. In 2021, Karvelis et al. [206] confirmed that TnpB, expressed by *Deinococcus radiodurans* ISDra2, is a programmable RNA‐guided nuclease and revealed that TnpB is the ancestor of Cas12. In 2022, Kazuki et al. [207] elucidated the structure of the IscB‐ωRNA RNP complex, guessing that it might be the ancestor of Cas9. Shortly thereafter, Ning et al. [208] revealed the evolutionary relationship between IscB and Cas9. In the same year, Feng Zhang's team utilized cryo‐electron microscopy to reveal the three‐dimensional structure of the key component of the OMEGA system, IsrB (OMEGA nickase), in a complex with ωRNA and target DNA [209]. In 2023, Zhang Feng's team reported the discovery of the Fanzor (Fz) system in eukaryotes [210, 211]. The Fz system could be reprogrammed for human genome engineering applications. This work conclusively demonstrated that RNA‐guided nucleases are present across all three domains of life. More recently, Patrick Hsu's team [212, 213], in their research on *IS110* insertion sequences, discovered novel bridge RNAs that mediate a dual‐specificity DNA recognition mechanism. These bridge RNAs can simultaneously pair with target DNA and donor DNA through their internal loops, thereby guiding recombinases to specific gene sites. By independently reprogramming bridge RNA's target‐binding loop and donor‐binding loop, they achieved precise control over sequence‐specific recognition between dsDNA molecules, thereby enabling accurate reshuffling, insertion, and excision of large DNA fragments in the genome.

These newly discovered dsDNA targeting technologies expand the existing toolbox [212, 214, 215, 216, 217, 218, 219, 220, 221, 222], but their full potential as molecular tools requires further exploration. The miniaturization and high specificity of the OMEGA system demonstrate its promise for intracellular applications, while the bispecific recognition mechanism of Bridge RNA offers a novel approach to genome rearrangement. However, whether these systems can rival the CRISPR/Cas system in dominance depends on several factors: (1) Operational efficiency and specificity: Compared to CRISPR/Cas, the editing efficiency and off‐target effects of these new systems remain unclear. (2) Functional diversity: Current research on their target range and mechanisms of action is still limited. (3) Clinical feasibility: The stability and safety of these systems in complex organisms must be further validated. Overall, with continued technological advancements, these new systems have the potential to complement the CRISPR/Cas system and may even enable unique applications in specific fields. In‐depth future studies will likely uncover additional possibilities for gene editing and molecular diagnostics.

## 5 Conclusion and Outlook

dsDNA targeting technologies have demonstrated significant potential and serve as fundamental tools for gene detection and editing. This review summarizes the current dsDNA targeting technologies from denaturation‐dependent and denaturation‐independent strategies. We elucidate the mechanisms through which these systems recognize and bind to target genes, along with their diverse application scenarios. Particular attention is given to the research progress and practical applications of non‐denaturing systems, such as CRISPR/Cas, Ago systems, and the λ Exo‐pDNA system. Despite their considerable promise, these technologies encounter several challenges in practical implementation and require further optimization and breakthroughs to fully realize their potential.

From the necessity of denaturation:

1. Universality and limitations of denaturation strategies

DNA denaturation is essential in dsDNA targeting due to the double helix's stability, which limits nucleotide access. Thermal and chemical methods, widely used in clinical diagnostics for their effectiveness and compatibility with AI‐enhanced signal analysis, effectively enable probe access. However, they risk DNA damage, epigenetic marker changes, and impede real‐time monitoring of nucleic acid interactions.

2. Advantages and problems of denaturation‐free strategies

Denaturation‐independent strategies offer a milder, more specific, and more adaptable alternative. Avoiding high temperatures or chemical agents, they minimize DNA damage and epigenetic alterations while allowing real‐time monitoring in living systems, making them ideal for clinical diagnostics, in situ imaging, and gene editing. Yet, challenges like probe stability in complex biological environments and cytotoxicity in therapeutic applications remain to be considered.

From the perspective of gene detection:

3. Challenges with pre‐amplification

Most molecular assay systems require pre‐amplification of target sequences, although high‐sensitivity platforms like single‐molecule fluorescence and GFET are exceptions. While pre‐amplification improves detection sensitivity when combined with molecular probes, it is time‐consuming, prone to contamination, and increases the risk of false positives. To ensure accurate results, it is essential to optimize amplification parameters, including primer design, temperature control, amplification duration, and maintaining a contamination‐free environment.

4. Improving single nucleotide variant (SNV) detection

Although the CRISPR/Cas system has shown promise in detecting SNVs, research on emerging systems like Agos and the λ Exo‐pDNA system remains limited. Future studies should focus on these technologies to better understand their specificity for mutant bases and mutation sites, enabling further optimization and application in precise SNV detection.

5. Developing integrated detection systems

Integrated platforms that combine sample extraction, identification, and signal output are crucial to minimizing the complexity of genetic detection. These systems reduce handling time, human error, and operational challenges, making them ideal for point‐of‐care testing. Designing such platforms to align with the unique characteristics of various probes allows for fast, accurate, and user‐friendly testing beyond traditional laboratory environments. Innovations in automation, microfluidics, and AI‐driven analysis are essential to advancing these systems.

6. Overcoming imaging challenges in living cells

In situ imaging of genes within living cells remains a significant hurdle, especially for visualizing non‐repetitive sequences and conducting multiplex imaging. Current techniques are limited in their sensitivity and often struggle with interference from non‐specific signals. Advancements in molecular labeling, high‐resolution microscopy, novel imaging technologies, and powerful image recognition algorithms are critical to improving the localization of low‐abundance genes and enhancing signal clarity in live‐cell imaging.

From the perspective of gene editing:

7. Diversification and optimization of precision in gene editing tools

Gene editing tools have gradually evolved from ZFPs and TALENs to the widely used CRISPR/Cas systems, with the potential for additional tools to emerge. The research focus is shifting from merely enhancing editing capacity to improving editing efficiency and specificity.

8. Minimization of off‐target effects

The off‐target effects remain a critical issue in gene editing and can be reduced by optimizing targeting sequences, developing new nuclease variants, and incorporating correction mechanisms. Meanwhile, prediction models based on deep learning and artificial intelligence are emerging to predict off‐target sites, further enhancing the precision of gene editing.

From the AI‐integrated targeting technologies:

AI is transforming dsDNA technologies by enhancing the design of molecular tools, optimizing target recognition, and improving data analysis. Machine learning models are increasingly integrated into probe selection processes to improve binding efficiency and minimize off‐target effects [223, 224]. Additionally, AI‐driven molecular dynamics simulations aid in refining probe structures and assessing their stability under physiological conditions [88, 168]. Deep learning techniques further enable the extraction of meaningful patterns from genomic imaging data, facilitating real‐time monitoring of genetic modifications [225, 226]. These advancements are driving more precise and efficient dsDNA‐targeting technologies, paving the way for breakthroughs in gene editing, diagnostics, and therapeutics.

In conclusion, the continued advancement of dsDNA targeting technologies relies on multidisciplinary collaboration and the seamless integration of AI‐driven approaches with gene detection and editing tools. While these innovations hold great promise for precision diagnostics and therapeutic applications, challenges such as technical complexity, high costs, safety concerns, ethical considerations, and regulatory hurdles must be addressed. Overcoming these obstacles will require technological refinements, cost management strategies, rigorous safety assessments, ethical oversight, and well‐defined regulatory frameworks. Through these collective efforts, dsDNA targeting technologies can fully realize their potential to transform biomedical research and clinical practice.

## Conflicts of Interest

The authors declare no conflicts of interest. Huiyu Liu is a member of the *Exploration* editorial board, and she was not involved in the handling or peer review process of this manuscript.
